# Intravasation‐On‐µDevice (INVADE): Engineering Dynamic Vascular Interfaces to Study Cancer Cell Intravasation

**DOI:** 10.1002/adma.202501466

**Published:** 2025-04-13

**Authors:** Fengtao Jiang, Yingqi Zhang, Guocheng Fang, Yao Wang, Alexander Dupuy, Jasmine Jin, Yi Shen, Khoon S Lim, Yinyan Wang, Yu Shrike Zhang, Ann‐Na Cho, Hongxu Lu, Lining Arnold Ju

**Affiliations:** ^1^ School of Biomedical Engineering Faculty of Engineering The University of Sydney Darlington NSW 2008 Australia; ^2^ School of Electrical & Electronic Engineering Nanyang Technological University 50 Nanyang Avenue Singapore 639798 Singapore; ^3^ Charles Perkins Centre The University of Sydney Camperdown NSW 2006 Australia; ^4^ The University of Sydney Nano Institute (Sydney Nano) The University of Sydney Camperdown NS 2006 Australia; ^5^ School of Chemical and Biomolecular Engineering Faculty of Engineering The University of Sydney Darlington NSW 2008 Australia; ^6^ School of Medical Sciences The University of Sydney Darlington NSW 2008 Australia; ^7^ Department of Neurosurgery Beijing Tiantan Hospital Capital Medical University Beijing 100070 China; ^8^ The George Institute for Global Health Barangaroo 2000 Australia; ^9^ Division of Engineering in Medicine Department of Medicine Brigham and Women's Hospital Harvard Medical School Cambridge MA 02139 USA; ^10^ State Key Laboratory of High Performance Ceramics and Superfine Microstructure Shanghai Institute of Ceramics Chinese Academy of Sciences 1295 Dingxi Road Shanghai 200050 P. R. China; ^11^ Heart Research Institute Camperdown Newtown NSW 2042 Australia

**Keywords:** cancer metastasis, endothelium, epithelial‐mesenchymal transition, intravasation, microfluidics, vimentin

## Abstract

Cancer metastasis begins with intravasation, where cancer cells enter blood vessels through complex interactions with the endothelial barrier. Understanding this process remains challenging due to the lack of physiologically relevant models. Here, INVADE (Intravasation‐on‐µDevice), a biomimetic microfluidic platform, is presented, enabling high‐throughput analysis of cancer cell intravasation under controlled conditions. This engineered platform integrates 23 parallel niche chambers with an endothelialized channel, providing both precise microenvironmental control and optical accessibility for real‐time visualization. Using this platform, distinct intravasation mechanisms are uncovered: MCF‐7 cells exhibit collective invasion, while MDA‐MB‐231 cells demonstrate an interactive mode with three functionally distinct subpopulations. A previously unknown epithelial‐mesenchymal transition (EMT) and mesenchymal‐epithelial transition (MET) switch is We discovered during intravasation, where MDA‐MB‐231 cells initially increase Vimentin expression before undergoing a 2.3 fold decrease over 96 h alongside a 1.5 fold increase in epithelial cell adhesion molecule (EpCAM). Remarkably, endothelial cells directly suppress cancer cell mesenchymal properties, as evidenced by a 4.6 fold reduction in Vimentin expression compared to mono‐cultures. Additionally, bilateral cancer‐endothelial interactions are revealed, aggressive cancer cells induce significant intercellular adhesion molecule‐1 (ICAM‐1) upregulation in endothelium. The INVADE platform represents an engineering advancement for studying complex cell–cell interactions with implications for understanding metastatic mechanisms.

## Graphical abstract

Cancer metastasis begins with intravasation, a complex process involving cancer‐endothelial interactions. INVADE (Intravasation‐on‐µDevice), a biomimetic microfluidic platform enables high‐throughput analysis of intravasation under controlled conditions. This system reveals distinct invasion modes, an epithelial‐mesenchymal transition (EMT) ‐ mesenchymal‐epithelial transition (MET) switch, and endothelial suppression of mesenchymal traits. It also uncovers bilateral signaling, highlighting dynamic cancer‐endothelial crosstalk with implications for metastasis research.

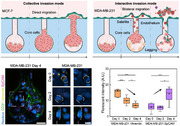

## 1 Introduction

Cancer metastasis remains the leading cause of cancer‐related mortality, accounting for ≈90% of deaths among cancer patients.^[^ ^1^ ^]^ Metastasis involves the dissemination of cancer cells from the primary tumor to distant organs via intravasation—where cancer cells transmigrate across the endothelial barrier to enter the bloodstream—serving as a critical early step in this process.^[^ ^2^, ^3^ ^]^

The complexity of cancer cell intravasation stems from intricate bilateral interactions between cancer cells and the endothelium.^[^ ^3^, ^4^, ^5^ ^]^ Traditional studies have identified key molecular players, including adhesion molecules (e.g. selectins and integrins) and signaling pathways that mediate cancer‐endothelial interactions.^[^ ^6^, ^7^, ^8^ ^]^ However, emerging evidence highlights the critical role of microenvironment in regulating intravasation efficiency.^[^ ^9^, ^10^ ^]^ The vascular microenvironment presents unique mechanical challenges, including fluid shear stress (0.5–10 dyne cm^−^^2^), varying vessel geometries, extracellular matrix stiffness, and endothelial barrier mechanics, all of which significantly influence cancer cell behavior.^[^ ^11^, ^12^, ^13^ ^]^

Moreover, different cancer types exhibit distinct metastatic potentials, indicating that cell type‐specific mechanisms govern intravasation.^[^ ^3^, ^14^, ^15^ ^]^ For instance, breast cancer cells such as MDA‐MB‐231 and MCF‐7 demonstrate markedly different invasive behaviors in vivo.^[^ ^16^ ^]^ Each cancer type, with unique biological properties and migratory patterns, directly impacts the metastatic potential, clinical outcomes, and treatment responses. Recognizing these differences enables more precise interventions, personalized treatment approaches, and better prognostic assessments.

More importantly, the epithelial‐mesenchymal transition (EMT), a key process in metastasis that drives cell migration, invasion, and the formation of secondary tumors, remains incompletely understood due to its multifaceted and dynamic nature. EMT involves a series of tightly regulated and reversible steps that are influenced by genetic, molecular, and environmental factors. Despite extensive research, significant gaps persist in understanding how tissue context, cellular heterogeneity, and mechanical cues in the microenvironment contribute to the initiation, progression, and differentiation of cancer cell subpopulations.^[^ ^4^, ^17^, ^18^, ^19^ ^]^

Current approaches for studying cancer intravasation face critical limitations. While in vivo models are physiologically relevant, they often lack the temporal and spatial resolution required to observe detailed cellular interactions.^[^ ^4^, ^20^ ^]^ Traditional *in‐* *vitro* systems, such as Transwell assays, fail to recreate the complex microenvironment of blood vessels and cannot fully capture dynamic cell–cell interactions.^[^ ^21^, ^22^ ^]^ Furthermore, these systems typically focus on endpoint analyses, missing crucial information about the temporal progression of cancer‐endothelial interactions.^[^ ^23^ ^]^

Recent advancements in microfluidic technology have led to the development of platforms that provide precise control over mechanical and chemical parameters, closely mimicking physiological conditions.^[^ ^13^, ^24^, ^25^ ^]^ However, many existing platforms focus primarily on the later stage of the metastasis cascade, neglecting intravasation as an early and critical event. Additionally, many of these platforms either lack the capability for high‐throughput analysis or fail to incorporate key features of the vascular microenvironment, such as controlled flow conditions and intact endothelial barrier establishment.^[^ ^26^, ^27^ ^]^ The potential existence of bilateral communication between cancer cells and endothelial cells has not been sufficiently investigated either.^[^ ^28^, ^29^ ^]^

To address these challenges, there is a pressing need for in‐vitro models that accurately recreate the vascular microenvironment while allowing high‐resolution, real‐time monitoring of the intravasation process.^[^ ^30^, ^31^ ^]^ Such platforms should integrate precise control over the tumor micro‐environment with the capability to monitor dynamic cell–cell interactions in real‐time while maintaining sufficient throughput for robust statistical analysis. Here, we present the INVADE (Intravasation‐on‐µDevice) platform—an innovative 3D microfluidic system designed specifically to investigate the real‐time cancer‐endothelial interactions during the intravasation process under physiologically relevant conditions.

## 2 Results

### 2.1 INVADE Platform Design and Implementation for High‐Throughput Cancer Intravasation Analysis

**Figure 1:**
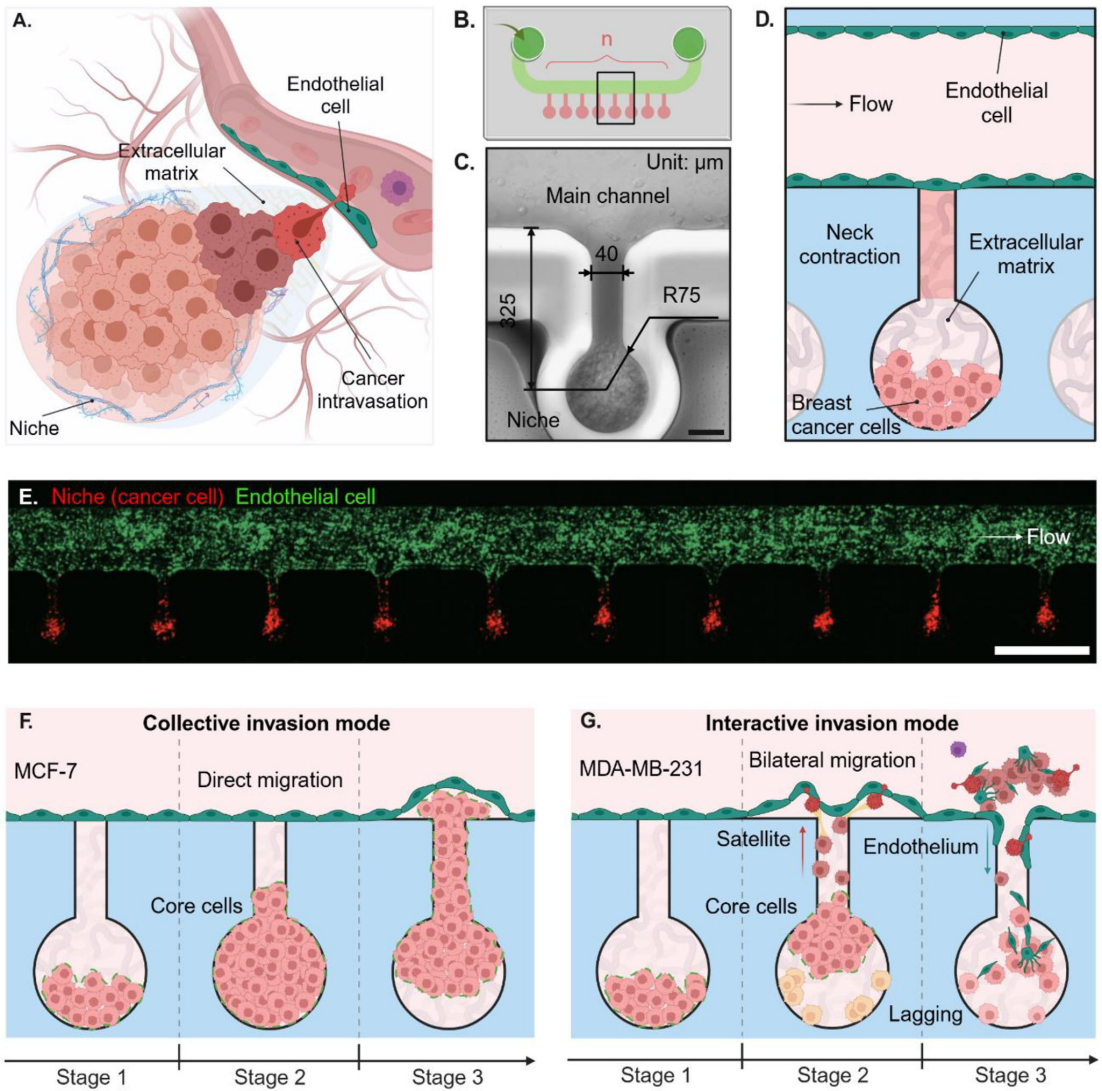
INVADE biomimetic microfluidic platform enabling high‐throughput analysis of cancer‐endothelial intravasation dynamics. A) Schematic of intravasation as the initial step in the metastasis cascade. B) Graphical illustration of a lab‐on‐chip device – INVADE to model intravasation of cancer cells to the vasculature. The INVADE platform integrates identical niche chambers (*n* = 23) with an endothelialized channel for high‐throughput analysis of cancer‐endothelial interactions. C) Zoom‐in image of an individual niche chamber connected to the endothelialized channel. Scale bar: 70 µm. D) Schematic of an individual intravasation unit showing main channels (endothelium residence and medium flow), niche chambers (cancer residence), and connecting necks (intravasation paths). E) Confocal image of the INVADE model co‐cultured with MDA‐MB‐231 breast cancer cells (red) and human umbilical endothelial cells (HUVEC, green) demonstrating efficient examination of cancer cell intravasation in a high‐throughput setup; scale bar: 500 µm. Conceptual illustration highlighting two distinct intravasation modes: F) collective invasion mode (MCF‐7) versus G) interactive invasion mode (MDA‐MB‐231). Light red cell: core cell; yellow cell: lagging cell; red cell: satellite cell; deep red cell: invading cancer cell; purple cell: escaped cancer cell green cell: endothelial cell. Illustration figures are created at https://BioRender.com.

To mimic the process of intravasation, we engineered a sophisticated microfluidic device that integrates multiple cancer cell niches with an endothelialized channel, enabling high‐throughput observation of cancer‐endothelial interactions (**Figure** **1**A–C; Figures S1 and S2, Supporting Information). Specifically, this novel microfluidic platform features an array of 23 identical niche chambers (radius: 75 µm) connected to the main channel through constriction necks (width: 40 µm, length: 255 µm, cross‐sectional area: 12 000 µm^2^) that impose contraction‐like confinement, mimicking the physical barriers encountered by cancer cells in vivo (Figure 1C). The main channels (width: 300 µm, height: 200 µm) were designed to recapitulate vasculature and maintain physiological flow conditions (shear stress: 0.5–10 dyne cm^−^^2^, Figure 1D; Figure S3, Supporting Information). This architecture provides physiologically relevant mechanical constraints during cancer cell intravasation while maintaining experimental consistency across multiple chambers, allowing for robust and reproducible studies of cancer cell dynamics under controlled conditions. The INVADE represents an innovative platform for studying cancer cell entry into blood vessels by replicating the in‐vivo environments encountered during intravasation.

To validate the INVADE platform's functionality, we performed co‐culture experiments using fluorescently labeled MDA‐MB‐231 breast cancer cells (red) and human umbilical vein endothelial cells (HUVECs, green). Confocal imaging demonstrated the successful establishment of the cancer niche and endothelium compartments, alone with their interfaces across multiple chambers simultaneously, confirming the platform's high‐throughput capability (Figure 1E). This configuration significantly increases experimental efficiency while maintaining consistent conditions across all observation units.

Using the INVADE platform, we revealed two distinct modes of intravasation between different breast cancer cell lines (Figure 1F,G). MCF‐7 cells exhibited a collective invasion mode, characterized by cohesive group migration (Figure 1F), while MDA‐MB‐231 cells demonstrated an interactive invasion mode featuring three distinct cell populations: core cells, satellite cells, and lagging cells (Figure 1G). Each subpopulation plays specific roles in the intravasation process, contributing to the complex dynamics of cancer cell entry into vessels.

### 2.2 Establishment of Stable Vessel‐Mimicking Microenvironment and Validation of Endothelial Barrier Functionality

**Figure 2:**
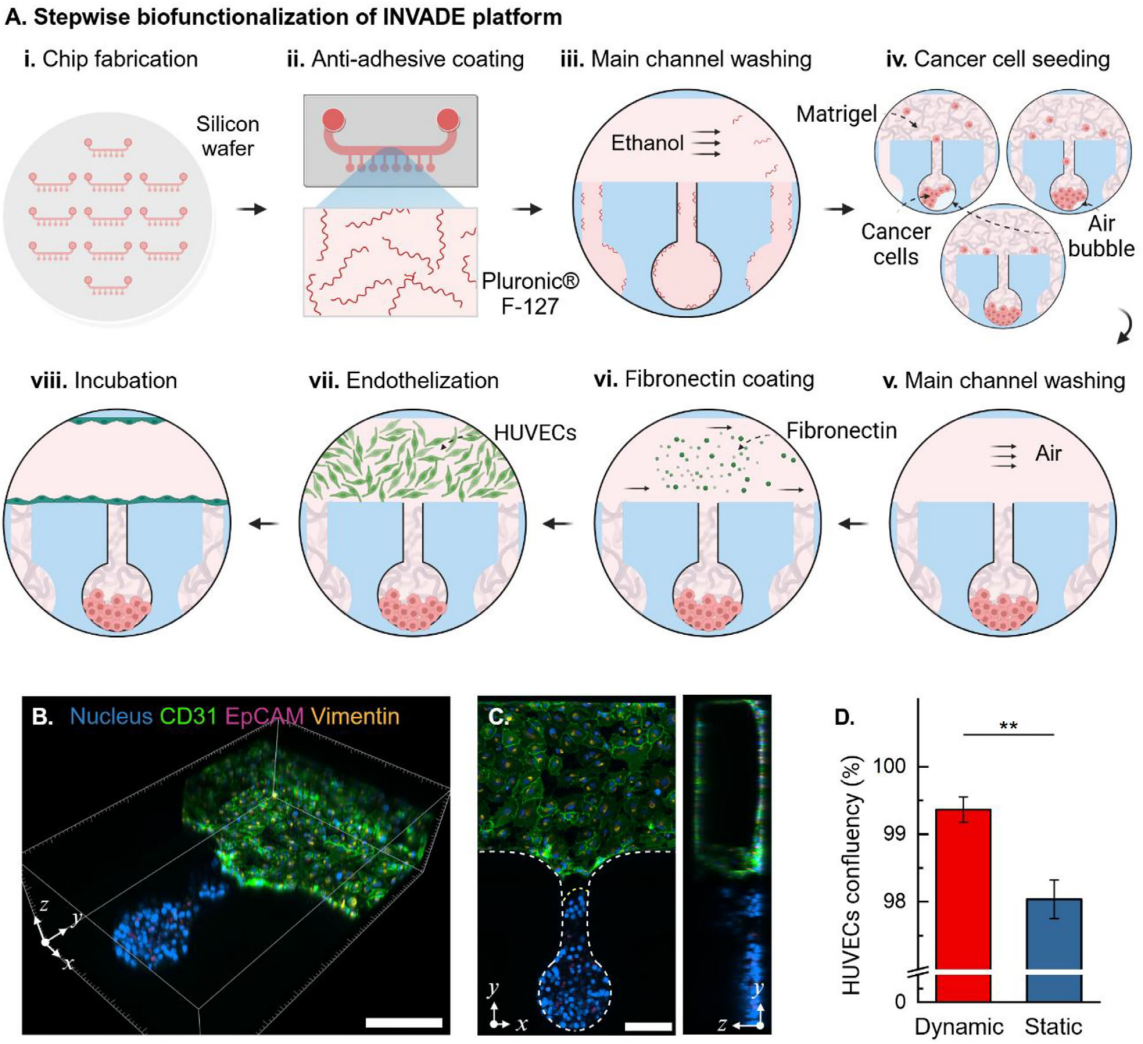
Biofunctionalization strategy establishes INVADE biomimetic microfluidic platform with a functional endothelial barrier. A) Stepwise demonstration of INVADE platform preparation for cancer‐endothelial interaction studies. i) Fabrication of INVADE microfluidic platform via soft lithography. ii, iii) Pluronic F‐127 treatment for non‐adhesive niche chamber to inhibit cancer cell adhesion and promote spheroid formation. iv) Cancer cell and Matrigel mixture seeding into the niche chamber. v) Removal of residual cancer cells and Matrigel mixture from the main channel. vi) Fibronectin coating of the main channel to support endothelial cell attachment. vii) Endothelialization of the main channel with human umbilical endothelial cells (HUVECs). viii) Formation of cancer cell niche and confluent endothelium within the INVADE platform. Confocal images of the B) three‐dimensional view, C) top view (left), and side view (right) of the INVADE platform cultured on Day 2. Scale bars: 200 µm (B), 100 µm (C). D) Comparison of HUVEC confluency under dynamic versus static culture conditions. Data are mean ± SEM. Statistical significance assessed by One‐Way ANOVA (^**^ *p* < 0.01; *n* ≥ 3). Illustration figures are created at https://BioRender.com.

The development of a stable vessel‐mimicking microenvironment in the INVADE platform required a meticulously optimized multi‐step fabrication and functionalization protocol (**Figure** **2**A). Initial device fabrication employed standard soft lithography techniques to create precise microchannel arrays essential for vascular architecture mimicry. A critical innovation in our protocol was the selective surface modification approach, where Pluronic F‐127 coating was specifically applied to the niche chamber while maintaining the main channel's capacity for endothelial cell adhesion (Figure 2A,ii,iii).

Prior to cell seeding, devices underwent vacuum treatment (>30 min) to eliminate air bubbles and ensure uniform surface modification. Cancer cells were perfused into the microchannel in the form of a specialized mixture of cell suspension and Matrigel (2:1 volume ratio), carefully optimized to recreate the complex extracellular environment containing laminin, collagen IV, and entactin/nidogen (Figure 2A,iv). Following a 15 min incubation on ice for bubble absorption, cells were centrifuged (2000 rpm, 30 s) to achieve uniform distribution at the chamber bottom. Afterwards, the device was placed in the incubator at 37 °C for 30 min for Matrigel gelation, which allowed the fixation of cancers at the niche chamber.

A crucial innovation in our protocol was the implementation of a repeated Matrigel seeding step after Matrigel gelation, specifically designed to fill the neck constriction and provide support for endothelial attachment to establish a clear endothelial barrier. Residual cells or Matrigel in the main channel were then carefully removed using airflow to maintain the integrity of the separate compartments after 30 mins of incubation at 37 °C (Figure 2A,v). Then 100 µg mL^−1^ fibronectin was introduced to allow selective coating of the main channel to promote endothelial cell attachment (Figure 2A,vi). Lastly, HUVECs suspension at 1 × 10^6^ cells mL^−1^ was perfused into the main channel (Figure 2A, vii) and allowed to attach on the channel surface for 30 min (Figure 2A, viii).

The endothelialization process resulted in the formation of a stable, confluent monolayer (Figure 2B,C), achieving over 99% coverage under dynamic culture conditions (Figure 2D). Three‐dimensional confocal microscopy confirmed the successful establishment of distinct compartments and clear visualization of the cancer‐endothelial interface (Figure 2B). Comparative analysis between dynamic and static culture conditions revealed significantly enhanced endothelial coverage under flow conditions (*p* < 0.001), highlighting the importance of mechanical stimulation for maintaining endothelial integrity (Figure 2C; Figures S4 and S5, Supporting Information).

To validate the integrity and functionality of the endothelial barrier, we performed confocal imaging at different z‐positions (50, 100, 150, and 200 µm) along the neck region (Figure S5, Supporting Information). These images confirmed the formation of a continuous endothelial monolayer at the neck‐main channel interface, with intact cell–cell junctions, as evidenced by the presence of CD31 staining. Additionally, we conducted diffusion experiments using 3‐ to 5‐kDa FITC‐Dex to assess barrier permeability (Figure S6, Supporting Information). In the absence of an endothelial layer, FITC‐Dex rapidly diffused into the niche chambers within 30 s. Conversely, the presence of a confluent endothelial layer significantly restricted diffusion even after 60 s, demonstrating effective barrier function. Notably, stimulation with PMA (50 ng mL^−1^) did not result in a substantial change in diffusion, suggesting the minimal impact of PMA on endothelial permeability.

### 2.3 Cancer Type‐Specific Migration Patterns During Intravasation

**Figure 3:**
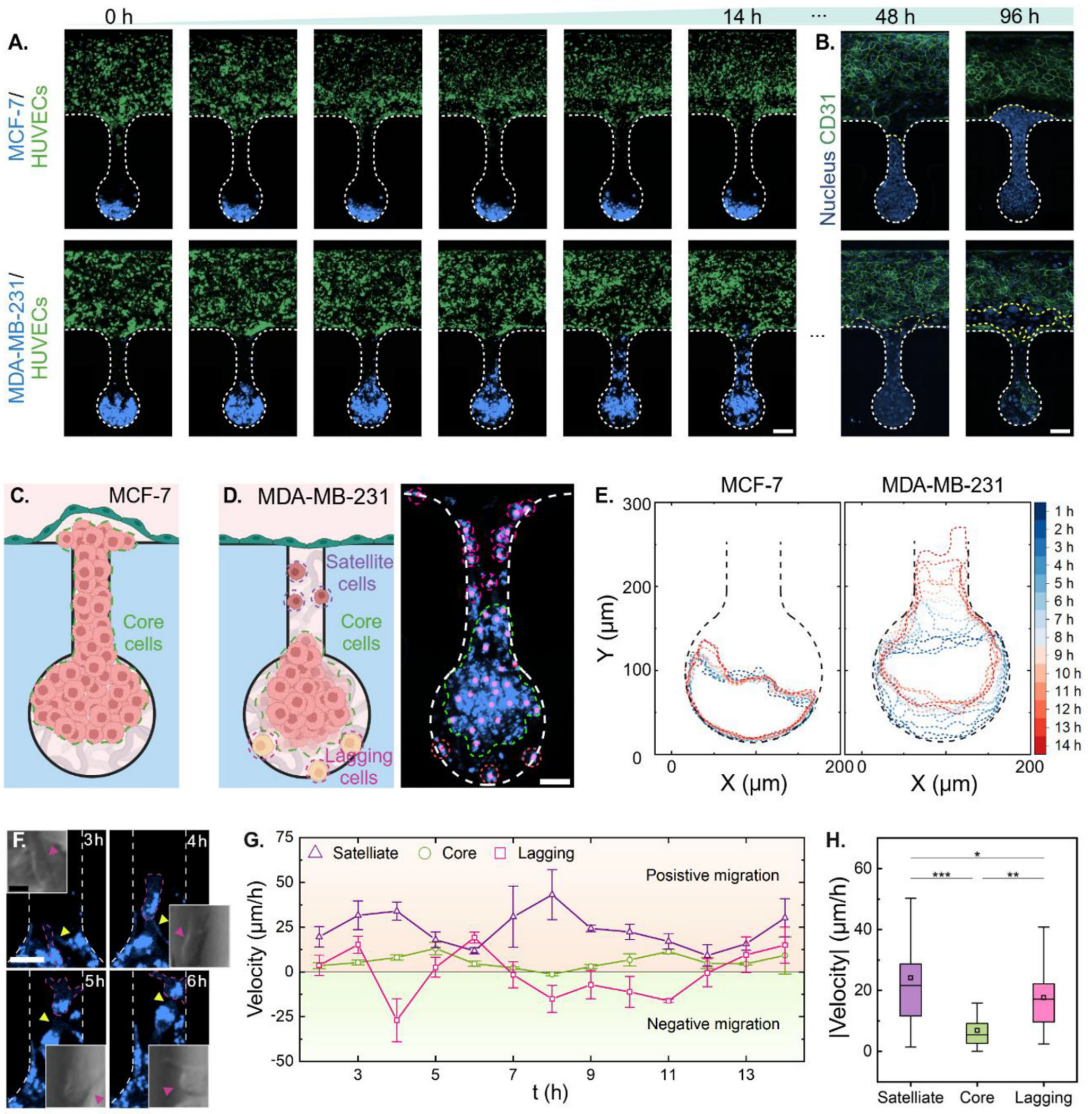
Distinct migratory behaviors of MCF‐7 and MDA‐MB‐231 during intravasation. A, B) Time‐lapse imaging demonstrates distinct migration modes of MCF7 (collective invasion) versus MDA‐MB‐231 (interactive invasion). HUVECs were labeled with PKH67, while MCF7 and MDA‐MB‐231 were labeled with PKH26 in panel (A). Hoechst 33342 and CD31 were used in panel (B) to label the nucleus and endothelium. Scale bar: 70 µm. C) MCF7 cells display uniform collective migration. D) MDA‐MB‐231 cells exhibit three distinct populations: satellite cells (leading edge penetration), core cells (progressive margination), and lagging cells (stationary). E) Contour profiles of cancer cells highlighting the distinct migratory behaviors of MDA‐MB‐231 and MCF7 during intravasation. F) Representative immunofluorescent images showing morphological transformations of MDA‐MB‐231 cells over 3–6 h. Scale bars: 40 and 15 µm (zoomed‐in view). G) Velocity profiles for three different cell populations of MDA‐MB‐231 from 0 to 14 h. H) Mean velocity of MDA‐MB‐231 from 0 to 14 h. Data are presented as mean ± SEM in panel (G). Box plots in (H) indicate the median (middle line), mean (square), the first and third quartiles (box), and the 10th and 90th percentile (error bars) of the velocity. Statistical significance was assessed using One‐Way ANOVA (^*^ *p* < 0.05; ^**^ *p* < 0.01; ^***^ *p* < 0.001; *n* ≥ 3). Illustration figures are created at https://BioRender.com.

Time‐resolved imaging using dual fluorescent labeling (PKH67 for HUVECs, PKH26 for cancer cells) revealed fundamentally different invasion modes between two breast cancer cell lines, MCF‐7 and MDA‐MB‐231 cells (**Figure** **3**A,B). MCF‐7 cells exhibited collective invasion behavior, maintaining cell–cell contacts throughout the migration process (Figure 3C). In contrast, MDA‐MB‐231 cells demonstrated an interactive invasion mode characterized by distinct cell populations (Figure 3D). Detailed analysis revealed three MDA‐MB‐231 subpopulations with unique behaviors (Figure 3D): satellite cells, named for their behavior similar to satellites as they guide subsequent cells throughout intravasation by acting as pioneers to clear the pathway and making initial contact with the endothelial barrier; core cells, which show coordinated margination; and lagging cells, named for their position deep within the niche.^[^ ^32^ ^]^ Cancer cell contour profiling quantified these distinct migratory patterns (Figure 3E).

Single‐cell morphology analysis of MDA‐MB‐231 cells during 3–6 h of invasion showed distinct morphological transitions during a relatively short response time (<6 h) (Figure 3F). From 3–4 h, the pink dotted line labeled cell adopted an elongated, spindle‐like morphology, characteristic of the mesenchymal phenotype, enabling it to penetrate dense ECM regions. This elongation facilitates movement through narrow spaces within the matrix. At 5‐h time mark, the cell transformed into an “octopus‐like” structure, extending multiple protrusions or filopodia. These extensions increase the cell's surface area, enhancing its ability to sense and respond to environmental cues, such as chemotactic signals or variations in ECM stiffness. At 6 h time mark, the cell exhibited a “snail‐like” morphology, characterized by a polarized shape with a broad leading edge and a contracted rear. This configuration is optimized for amoeboid movement, allowing rapid translocation and efficient navigation through the ECM.^[^ ^33^ ^]^ These morphological adaptations are indicative of cell plasticity and their ability to switch between different modes of migration in response to microenvironmental cues.

Further, velocity profiling demonstrated significant differences between subpopulations (Figure 3G,H), with satellite cells showing the highest motility (24.1 µm h^−1^) compared to core cells (6.7 µm h^−1^) and lagging cells (17.6 µm h^−1^). The observed morphological transformations and sub‐population‐dependent velocity profile suggest that MDA‐MB‐231 cells possess a high degree of phenotypic plasticity, enabling them to adapt their migration strategies during intravasation. This adaptability is a hallmark of aggressive cancer cells and is associated with increased metastatic potential. By modulating their shape and migratory mechanisms, these cells can effectively traverse the ECM, breach endothelial barriers, and enter the circulatory system, thereby facilitating the dissemination to distant sites.^[^ ^34^ ^]^ The INVADE platform provides the observation window for the complex interplay of intracellular signaling pathways and interactions within the ECM, including cytoskeletal remodeling,^[^ ^35^, ^36^ ^]^ extracellular matrix interactions,^[^ ^37^, ^38^ ^]^ signaling pathways,^[^ ^39^ ^]^ etc.

### 2.4 Biphasic EMT‐MET Dynamics in Cancer Cells and Endothelial Regulation

Epithelial–mesenchymal dynamics is a biological process where epithelial cells acquire mesenchymal characteristics, enhancing their migratory and invasive capabilities, which is pivotal in cancer progression, particularly during intravasation—the entry of cancer cells into the bloodstream—a critical step in metastasis.^[^ ^1^, ^3^, ^35^ ^]^ In our study, we investigated the dynamic expression patterns of Vimentin and EpCAM during intravasation through different time scales.

**Figure 4:**
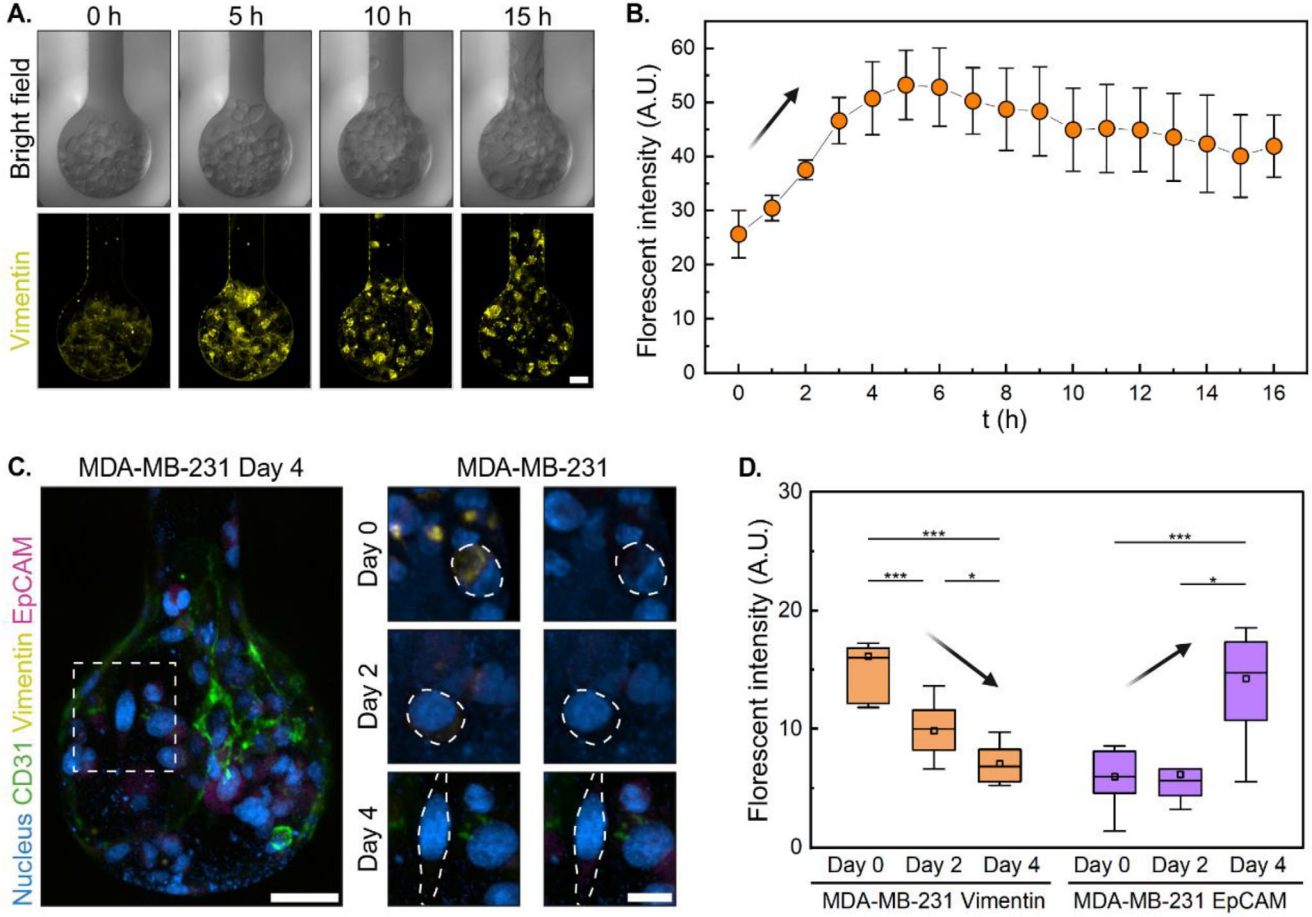
Biphasic EMT‐MET dynamics of MDA‐MB‐231 during intravasation. A) Live cell imaging demonstrates distinct Vimentin variation in MDA‐MB‐231 cells during the early intravasation phase (0–16 h). Scale bars: 30 µm. B) Quantitative analysis of Vimentin intensity through cancer‐endothelial co‐culture from 0 to 16 h, showing initial upregulation followed by decrease. C) Left: Epithelial‐mesenchymal transition markers (Vimentin, 1:3000, yellow; EpCAM, 1:400, purple) of MDA‐MB‐231 co‐cultured with HUVECs on day 4. Right: Time‐resolved evolution of Vimentin (Yellow, *left*) and EpCAM (Purple, *right*) in MDA‐MB‐231 cells over 4 days. Scale bars: 100 µm (left) and 20 µm (right). Time‐resolved evolution of epithelial‐mesenchymal transition markers (Vimentin, 1:3000, yellow; EpCAM, 1:400, purple) in MDA‐MB‐231 cells over longer timeframes (0–4 days). Scale bars: 100 µm (left) and 20 µm (right). D) Quantitative analysis of EMT marker expression showing an inverse relationship between Vimentin and EpCAM expression over time, indicating MET after initial EMT. Data are presented as arbitrary units (A.U.). Box plots in (D) indicate the median (middle line), mean (square), the first and third quartiles (box), and the 10th and 90th percentile (error bars) of the intensity. Data are presented as mean ± SEM. Statistical significance was assessed using One‐Way ANOVA (^*^ *p* < 0.05; ^**^ *p* < 0.01; ^***^ *p* < 0.001; *n* ≥ 5). Vimentin intensity was quantified using ImageJ from at least 30 cells per timepoint.

Our analysis revealed a biphasic pattern of mesenchymal marker expression in MDA‐MB‐231 cells during intravasation. Time‐resolved imaging over the initial 16 h showed that Vimentin expression first increased during the early phase of invasion (**Figure** **4**A,B). Initially, the Vimentin signal was at a relatively low expression level with a quantitative value of 26 (A.U.). From 0 to 5 h, the Vimentin intensity reached its peak value at 53 (A.U.), representing an early EMT phase that promotes initial invasive capabilities. During this phase, MDA‐MB‐231 cells exhibited enhanced mesenchymal traits, enabling them to navigate through the stromal environment toward the vasculature.

**Figure 5:**
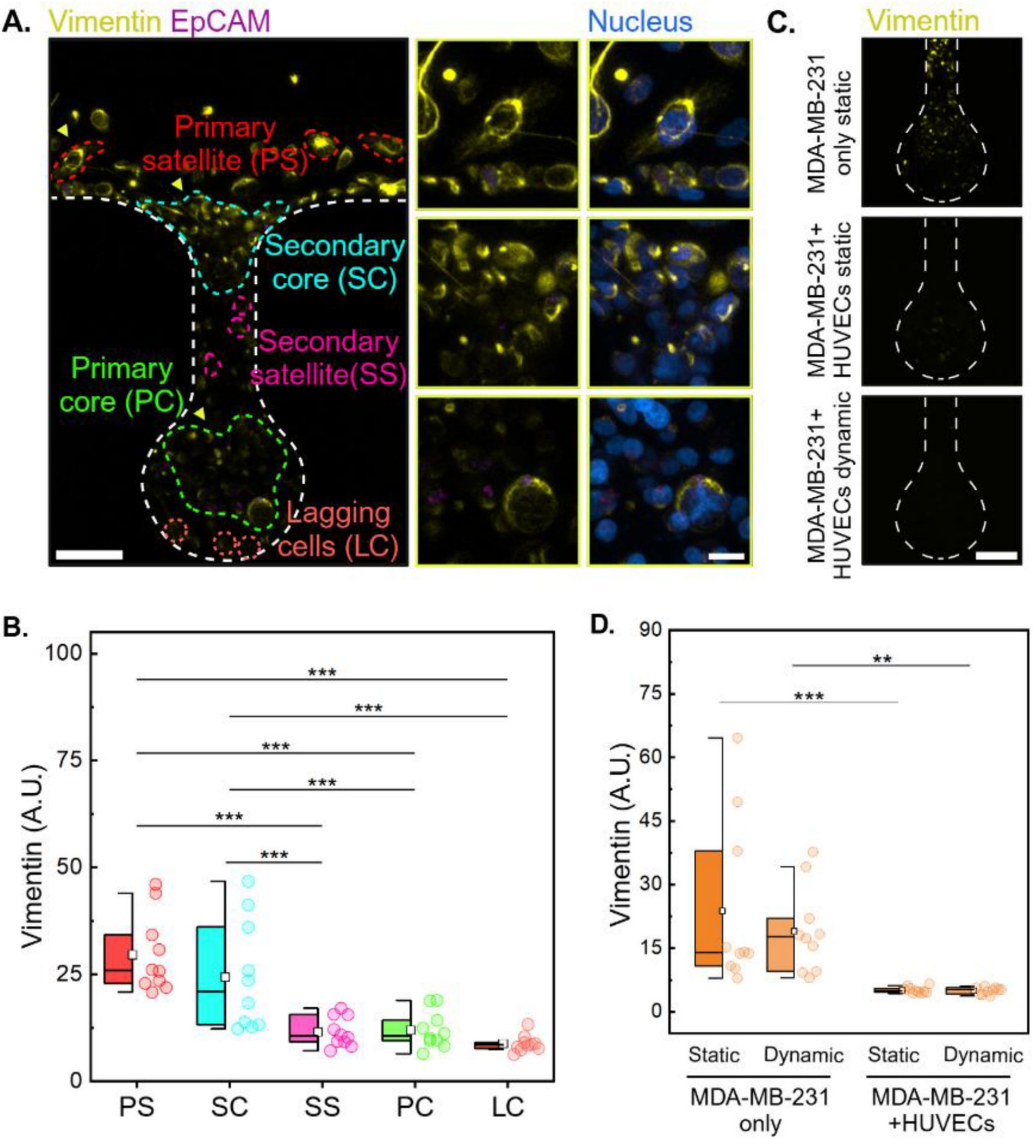
Sub‐population‐dependent cancer cell mesenchymal marker expression and endothelial inhibitory effect. A) Mapping of MDA‐MB‐231 mesenchymal marker (vimentin) expression across five distinct cell populations: Lagging cells (LC), primary core (PC), secondary satellite (SS), secondary core (SC), and primary satellite (PS), under dynamic culture conditions without HUVECs for two days. The yellow boxes indicate zoomed‐in regions, as pointed to by the yellow arrows. Scale bars: 70 µm (left) and 20 µm (right). B) Quantitative analysis of vimentin expression across different populations of MDA‐MB‐231. C) Vimentin expression under three other conditions: MDA‐MB‐231 only static culture, MDA‐MB‐231+HUVECs static co‐culture, and MDA‐MB‐231+HUVECs dynamic co‐culture. Scale bar: 70 µm. D) Quantitative analysis of Vimentin expression across different conditions. Box plots in (B, D) indicate the median (middle line), mean (square), the first and third quartiles (box), and the 10th and 90th percentile (error bars) of the intensity. Statistical significance was determined by One‐Way ANOVA or Two‐Way ANOVA (^**^ *p* < 0.01; ^***^ *p* < 0.001; *n* ≥ 5).

Interestingly, after this initial peak, Vimentin expression began to slightly decrease from 5 to 16 h, stabilizing ≈40 (A.U.) (Figure 4B). Following that, a further declining trend continued over a longer time scale (within 4 days), with our analysis over 96 h revealing a progressive 2.3 fold decrease in Vimentin expression by day 4 (Figure 4C,D). Concurrently, EpCAM, an epithelial marker, exhibits a 1.5 fold increase within the same timeframe (Figure 4C,D). This inverse relationship suggests a partial mesenchymal‐epithelial transition (MET), potentially reducing the metastatic propensity of MDA‐MB‐231 cells after contacting endothelial cells and promoting cell–cell adhesion and proliferation, which are essential for establishing new tumor colonies. On the contrary, MDA‐MB‐231 cells alone did not show such MET transition. There was an enhanced Vimentin expression over 96 h (4.6 fold and 3.8 fold difference, *p* < 0.001 and *p* < 0.01, for static and dynamic culture, respectively) of MDA‐MB‐231 cells alone compared to HUVECs co‐culture group (**Figure** **5**A,C,D; Figures S7 and S8, Supporting Information). This suggests that MDA‐MB‐231 cells present no tendency toward MET in the absence of endothelial cells. In short, the results indicate that the presence of HUVECs plays a significant role in the modulation of the epithelial‐mesenchymal transition process, specifically in MDA‐MB‐231 cells.

**Figure 6:**
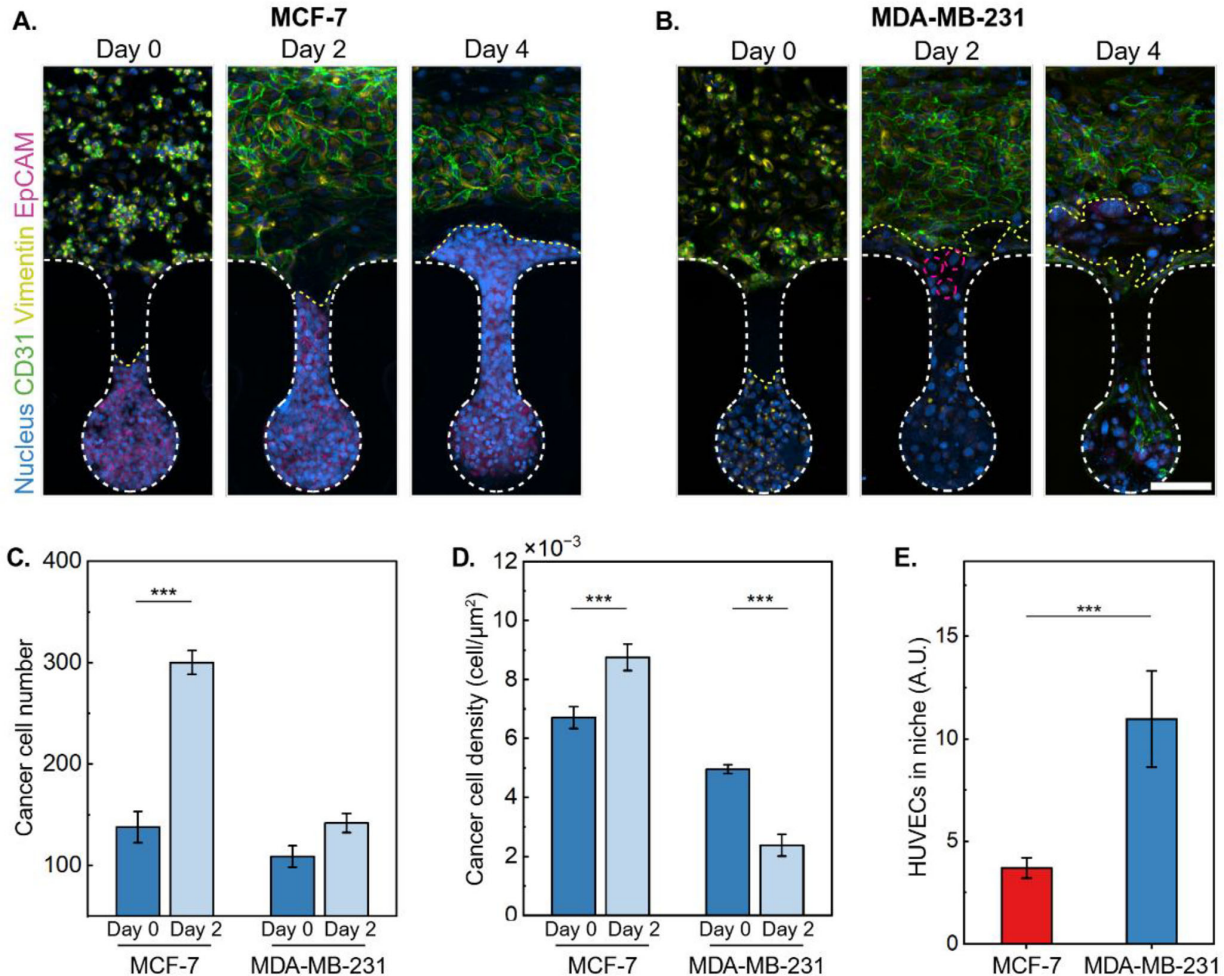
Cancer type‐specific proliferation and bilateral cancer‐endothelial interactions. Time‐resolved imaging reveals two distinct communication patterns between A) MCF‐7 and B) MDA‐MB‐231 co‐cultured with endothelial cells over 4 days. CD31 (1:400, green) and Hoechst 33342 (1:1000, blue) were used to label endothelium and nuclei. Pink dotted line indicates the satellite cells. Yellow dotted line indicates the MDA‐MB‐231 cells that migrated into the main channel. Scale bar: 100 µm. Quantitative analysis of C) cancer cell number and D) cancer cell density (cells µm^−^^2^), showing different proliferation and spatial organization patterns between the two cancer types. E) Quantitative analysis of cancer‐endothelial interactions on day 4, measured by CD31 intensity in niche chambers, showing 3 fold higher endothelial attraction by MDA‐MB‐231 compared to MCF‐7 (*p* < 0.001). Data presented as arbitrary units (A.U.). Data in (C–E) are expressed as mean ± SEM from at least 3 niche chambers per condition. Statistical significance was determined by One‐Way ANOVA or Two‐Way ANOVA (^***^ *p* < 0.001; *n* ≥ 3). Cell density was calculated by dividing cell number by the measured area occupied within each niche.

In contrast, MCF‐7 cells maintained low Vimentin expression from day 0 to day 4, while EpCAM expression remained consistently high during the same period (**Figure** **6**A). This indicates that MCF‐7 cells retain their epithelial characteristics, suggesting a lower invasive potential compared to MDA‐MB‐231 cells.

Moreover, in the absence of endothelial cells, MDA‐MB‐231 cells exhibited an advanced invasion pattern. Their distribution from the niche bottom to the main channel can be further categorized into five sub‐populations based on morphology and Vimentin expression (Figure 5A,B). Notably, cancer cell migration without endothelial interactions displays a distinct pattern in stage 3, differing from the co‐culture system. It is hypothesized that during the transition from stage 2 to stage 3, part of the original satellite cells transforms into a high Vimentin‐expressing sub‐population, termed primary satellites. These cells exhibit extensive filamentous extensions, forming connections with neighboring cells, indicative of enhanced migratory capacity and intercellular communication. The secondary core likely forms through the migration and amalgamation of the original satellite and core cells. Constrained by geometry, migration is impeded at the top neck region, promoting collective behavior and secondary core formation. The primary core formation may result from neck contraction, permitting only a portion of the cell cluster to migrate. Secondary satellite cells may function as information transmitters, facilitating communication between cores. Quantitative analysis reveals that primary satellite cells express vimentin at levels 2.5‐fold higher than primary core cells (Figure 5B and *p* < 0.001), indicating a spatial organization of EMT progression during intravasation.

Such dynamic modulation of the epithelial–mesenchymal markers underscores the plasticity of cancer cells in response to microenvironmental cues.^[^ ^40^ ^]^ Our findings highlight the importance of EMT and MET in cancer cell intravasation and subsequent metastatic colonization. Understanding these processes is crucial for developing targeted therapies aimed at inhibiting metastasis by epithelial–mesenchymal transition dynamics.

### 2.5 Cancer Type‐Specific Proliferation and Bilateral Endothelial Interactions

We observed distinct communication patterns between cancer and endothelial cells (Figure 6A,B), with particular emphasis on cancer cell proliferation and density dynamics (Figure 6C,D). MCF‐7 cells exhibit a substantial increase in cell number, rising from ≈138 cells on day 0 to ≈300 cells by day 2 (Figure 6A,C). Correspondingly, cell density escalates from 671 to 875 cells µm^−2^, indicating robust proliferative activity and spatial expansion (Figure 6D). In contrast, MDA‐MB‐231 cells show a modest increase in cell number, from 109 on day 0 to 142 on day 2 (Figure 6B,C). Notably, their cell density decreases from 496 to 238 cells µm^−2^, suggesting a propensity for dispersal rather than localized proliferation (Figure 6D).

These observations align with the inherent characteristics of the two cell lines. MCF‐7 cells, known for their epithelial‐like properties, tend to form cohesive clusters, leading to increased cell density as they proliferate. Conversely, MDA‐MB‐231 cells, which exhibit mesenchymal traits, are more migratory and invasive, often spreading out and reducing local cell density despite an increase in absolute cell numbers.

Furthermore, distinct interaction patterns between cancer cells and endothelial cells at the invasion interface were identified. Notably, MDA‐MB‐231 cells demonstrated enhanced endothelial attraction compared to MCF‐7 cells on day 4, exhibiting a threefold increase in interaction frequency (*p* < 0.001, Figure 6B, E; Figure S9, Supporting Information). This increased interaction was possibly due to the compromised endothelium and Matrigel matrix, which suggests a more aggressive interaction between MDA‐MB‐231 cells and the endothelial cells, which may facilitate tumor progression and metastasis. This bilateral migration pattern between MDA‐MB‐231 and endothelial cells closely resembles cancer cell‐induced angiogenesis observed in vivo^[^ ^41^, ^42^ ^]^ and clinically, such as in patients with GBM^[^ ^43^ ^]^ and pancreatic cancers,^[^ ^44^ ^]^ where aggressive cancer cells attract endothelial cells to form new vessels toward the tumor mass. This behavior may reflect the well‐documented secretion of pro‐angiogenic factors by MDA‐MB‐231 cells,^[^ ^42^, ^45^ ^]^ which can stimulate endothelial cell migration.

### 2.6 Differential Endothelial Remodeling and Functional Responses to Cancer Cells

**Figure 7:**
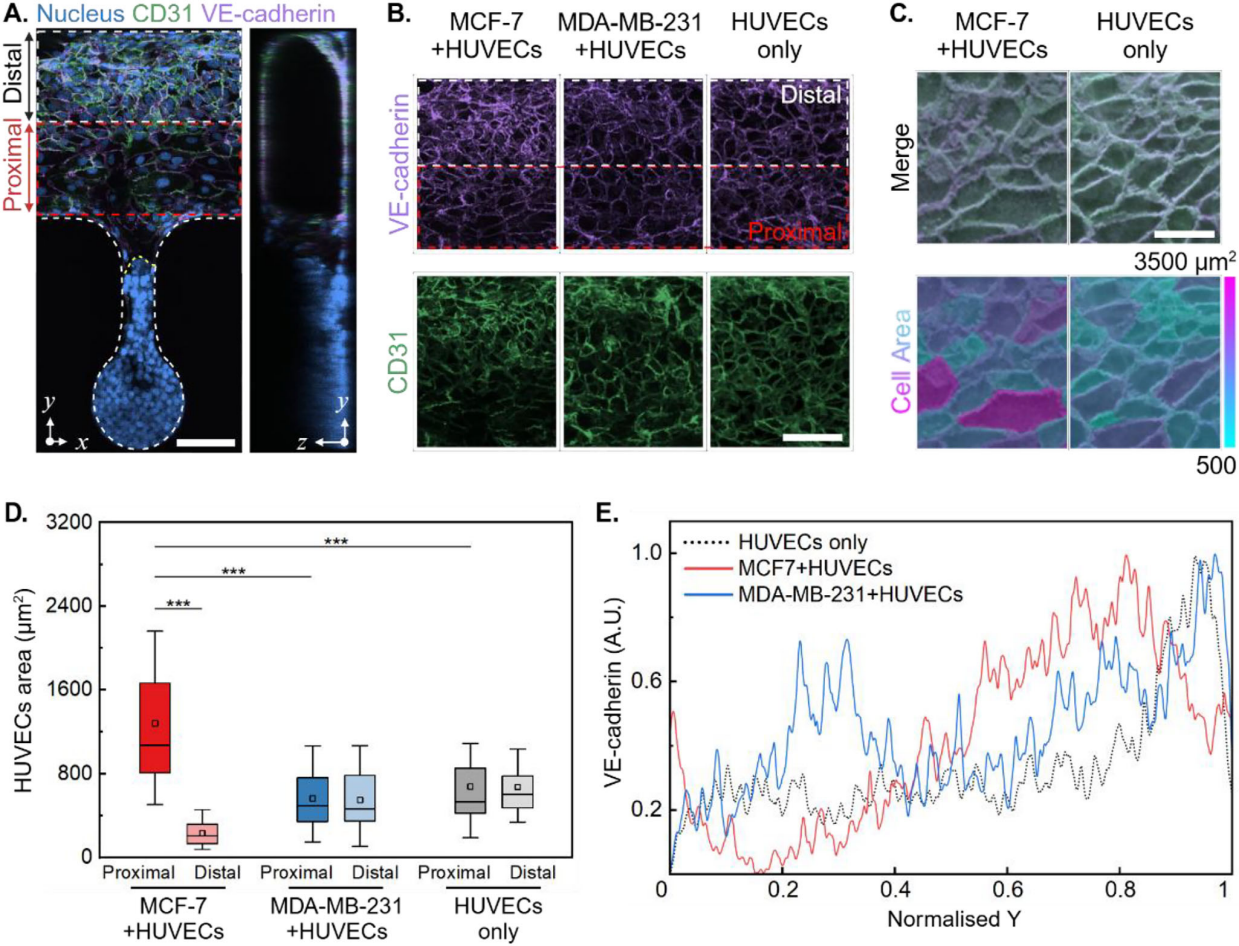
Differential endothelial remodeling responses to cancer cell types. A) Spatial mapping of HUVECs and cancer cells under dynamic culture conditions (1.875 µL min^−1^, 48 h). The main channel is divided into distal and proximal regions. The yellow dotted line indicates the cancer cell‐endothelial cell boundary. Scale bar: 100 µm. B) Confocal images of the endothelium in the main channel showing endothelial adherent junctions (VE‐cadherin, 1:400, purple) and platelet endothelial cell adhesion molecule (CD31, 1:400, green). Scale bar: 100 µm. C) Endothelial cell segmentation performed using the automated algorithm in ImageJ. Color bar: cell area from 500 to 3500 µm^2^ (indigo to magenta). D) Comparative analysis of HUVECs cell area (CA) distributions, showing significant morphological differences between proximal and distal regions in MCF‐7 co‐culture. E) VE‐cadherin intensity profile measured along main channel width, showing gradient patterns specific to each cancer type. Box plots in (E) indicate the median (middle line), mean (square), the first and third quartiles (box), and the 10th and 90th percentile (error bars) of the HUVECs CA. Statistical significance was determined by Two‐Way ANOVA (^***^ *p* < 0.001; *n* ≥ 30).

We investigated endothelial reorganization dynamics using two breast cancer cell lines under dynamic medium supply over a 48 h period (Experimental setup is presented in Figure S10, Supporting Information). Spatial mapping revealed distinct patterns of endothelial displacement, particularly evident when comparing proximal and distal regions relative to the cancer‐endothelial boundary (**Figure** **7**A; Figure S11, Supporting Information). The MCF‐7/HUVECs co‐culture showed a uniform displacement pattern, marked by a clear yellow dotted boundary line between cancer and endothelial cells.

Detailed analysis of endothelial adherens junctions (VE‐cadherin) and platelet endothelial cell adhesion molecule (CD31) in the main channel revealed cancer type‐specific reorganization patterns for proximal and distal regions. The cell bodies of the proximal HUVECs to the MCF‐7 cells were bigger and more elongated than the distal ones, which was not observed in HUVECs cocultured with MDA‐MB‐231 cells (Figure 7B). Cell segmentation analysis (color‐coded from 500–3500 µm^2^, indigo to magenta) demonstrated distinct morphological adaptations of endothelial cells in response to different cancer types (Figure 7C). The cell area distributions of HUVECs were analyzed in the presence of MCF‐7 and MDA‐MB‐231 cells. MCF‐7 cells induced a significant increase in endothelial cell area at the proximal region (mean area: 1279.58 µm^2^) and a decrease at the distal region (mean area: 230.93 µm^2^) (Figure 7D). In contrast, MDA‐MB‐231 cells resulted in a more uniform endothelial cell area distribution, with values ≈600 µm^2^, indicating a less pronounced effect on endothelial cell morphology compared to MCF‐7 cells (Figure 7D). Quantitative assessment of aspect ratio distributions showed significant differences between the MCF‐7/HUVECs co‐culture and HUVECs only (Figure S12A, Supporting Information) (*p* < 0.01).

Fluorescent intensity profiles of VE‐cadherin and CD31 along the main channel width revealed gradient patterns specific to each cancer type (Figure 7E; Figure S12B, Supporting Information). The MDA‐MB‐231/HUVECs co‐culture showed more pronounced junction reorganization in the proximal region compared to the MCF‐7/HUVECs co‐culture (3 fold difference).

**Figure 8:**
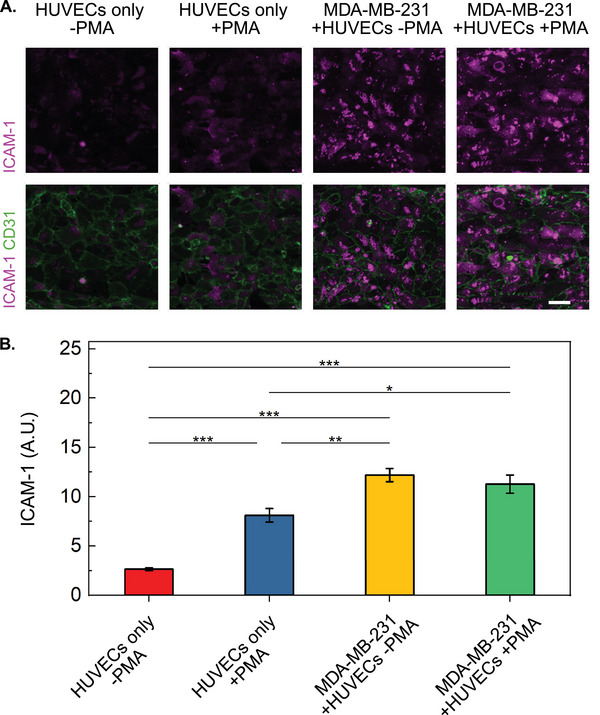
Endothelial ICAM‐1 expression in response to co‐culture with cancer cells and PMA stimulation. A) Fluorescent images of endothelium showing intercellular adhesion molecule 1 (ICAM‐1, 1:200, magenta) and platelet endothelial cell adhesion molecule (CD31, 1:300, green) in the main channel under four conditions: HUVECs only (±PMA) and HUVECs+MDA‐MB‐231 co‐culture (±PMA). PMA was applied at 50 ng mL^−1^ for 1.5 h before fixation. Scale bar: 50 µm. B) Quantitative analysis of ICAM‐1 expression across different conditions, showing significant upregulation in HUVECs upon PMA stimulation (3‐fold higher, p < 0.01) and even higher expression in the presence of MDA‐MB‐231 cells regardless of PMA treatment (5.5 fold higher, *p* < 0.001). Data are expressed as mean ± SEM from at least 3 regions of interest per condition. Statistical significance was determined by One‐Way ANOVA (^*^p < 0.05; ^**^ *p* < 0.01; ^***^ *p* < 0.001; *n* ≥ 3).

To further validate the functionality of the endothelium and examine the effect of cancer cells on endothelial activation, we investigated ICAM‐1 expression following PMA treatment under different culture conditions. The experiment consisted of four groups: HUVECs only with or without PMA treatment as well as the co‐culture of HUVECs and MDA‐MB‐231 cells with or without PMA treatment (**Figure** **8**A). The results demonstrated a significant upregulation of ICAM‐1 in HUVECs only upon PMA stimulation (3 fold higher, *p* < 0.01) (Figure 8A,B), confirming the responsiveness of our endothelial model to inflammatory stimuli. Remarkably, ICAM‐1 expression was even higher in the presence of MDA‐MB‐231 cells, regardless of PMA treatment (5.5 fold higher, *p* < 0.001) (Figure 8A,B).

This pronounced effect in the co‐culture conditions suggests that cancer cells strongly influence endothelial activation. MDA‐MB‐231 cells may induce ICAM‐1 expression, possibly due to the secretion of pro‐inflammatory cytokines such as TNF‐α and IL‐1β,^[^ ^46^, ^47^ ^]^ which are known to enhance endothelial adhesion molecule expression. Additionally, direct cell–cell interactions between cancer cells and endothelial cells could activate signaling pathways that upregulate ICAM‐1. Given that co‐culture lasted for 24 h, it is predicted that the sustained influence of cancer cell‐derived signals outweighed the transient effects of PMA stimulation (1.5 h). This finding highlights the critical role of tumor‐endothelial interactions in modulating endothelial function and adhesion molecule expression, which may have implications for cancer cell intravasation and metastasis.

## 3 Discussion, Conclusion, and Future Perspectives

The INVADE platform represents a significant advancement in studying cancer cell intravasation, providing unprecedented insights into the dynamic interactions between cancer cells and the endothelial barrier. Through this work, we have demonstrated four key findings: 1) the existence of distinct intravasation modes between different cancer cell types, 2) the critical role of endothelium‐regulated EMT‐MET dynamics during cancer intravasation, 3) the bilateral interaction between cancer cells and endothelial cells, and 4) the differential inflammatory response of endothelium to cancer cells.

Our comprehensive platform validation confirmed the establishment of a functional endothelial barrier with intact junction complexes before cancer‐endothelial interactions begin, as evidenced by FITC‐Dextran diffusion studies and z‐stack confocal imaging. The physiological responsiveness of endothelium within INVADE was further validated by ICAM‐1 upregulation following PMA stimulation, addressing concerns about endothelial barrier functionality at the critical neck‐channel interface.

Our results reveal fundamentally different intravasation strategies between MCF‐7 and MDA‐MB‐231 breast cancer cells. MCF‐7 cells exhibited collective invasion behavior, moving as a cohesive unit that physically expels the endothelial barrier. In contrast, MDA‐MB‐231 cells demonstrated a more sophisticated interactive invasion mode characterized by three distinct subpopulations: satellite cells, which act as pioneers in contacting the endothelium with the highest motility (24.1 µm h^−1^), core cells that maintain coordinated forward movement (6.7 µm h^−1^), and lagging cells that remain in the deep bottom of niche.^[^ ^14^, ^48^ ^]^

A particularly novel finding from our study is the dynamic regulation of EMT markers during intravasation. MDA‐MB‐231 cells co‐cultured with HUVECs showed a progressive 2.3 fold decrease in Vimentin expression accompanied by a 1.5 fold increase in EpCAM over 96 h, suggesting a partial MET. In stark contrast, MDA‐MB‐231 cells cultured without endothelial cells showed enhanced Vimentin expression with no tendency toward MET. This endothelial‐induced MET may represent an adaptive mechanism that facilitates the successful completion of the intravasation process.^[^ ^49^, ^50^ ^]^

When MDA‐MB‐231 cells were cultured independently of HUVECs, primary satellite cells exhibited the highest Vimentin expression, demonstrating a 2.5 fold increase compared to primary core cells. This spatial distribution highlights a hierarchical organization in EMT progression during cancer migration.^[^ ^51^ ^]^ To that end, while geometric constraints can influence cell migration patterns as demonstrated by Friedl et al.,^[^ ^52^ ^]^ our comparative analyses between cancer cells cultured with or without endothelial cells under identical geometrical conditions strongly suggest that the observed phenotypic changes in our current INVADE system are primarily driven by endothelial interactions rather than physical confinement.

The INVADE platform overcomes several limitations of existing approaches through its unique features: 1) high‐throughput capability (*n* = 23 parallel units) enabling robust statistical analysis while maintaining experimental consistency; 2) precise control over micro‐environment and geometries allowing investigation of programmable cancer‐endothelial interactions; 3) clear visualization of cellular dynamics through optimized optical properties and selective surface modification; 4) maintenance of distinct microenvironments through careful compartmentalization (>99% endothelial confluence under flow).

Last but not least, future studies should explorethe molecular mechanisms underlying cancer‐endothelial crosstalk and investigate how various geometrical constraints might influence cancer cell behaviors. To that end, future iterations of the INVADE platform could systematically vary confinement geometries to disentangle mechanical effects from biochemical signaling. These insights would further advance our understanding of cancer metastasis and suggest new therapeutic strategies targeting the cancer‐endothelial interface.

## 4 Experimental Section

### Microfabrication

The INVADE microfluidic device was fabricated using standard soft lithography techniques.^[^ ^53^, ^54^ ^]^ Briefly, a 200 µm constant height dry photoresist film (DJ micro laminate SUEX) was laminated on the silicon wafer at 85 °C to create the channel using a direct lithography writer (Heidelberg MLA‐100, 4500 mJ cm^−2^). When the lithography was finished, the wafer was taken for 5 min post‐baking at 90 °C, followed by PGMEA rinsing until the excess photoresist was completely removed. Polydimethylsiloxane (PDMS, Sylgard 184, Dow Corning) was mixed at a 10:1 ratio (w/w) of elastomer to curing agent, degassed, and poured over the master mold. After curing at 100 °C for 1.5 h, the PDMS layer was carefully peeled off, and access holes were punched using a 1.5 mm biopsy punch. The PDMS layer was bonded to the PDMS membrane using a plasma cleaner (Harrick Plasma).

### Surface Modification and Device Preparation

The INVADE platform underwent selective surface modification to create distinct microenvironments. Niche chambers were treated with Pluronic F‐127 (0.5% w v^−1^ in PBS) for 30 min at room temperature to prevent cell adhesion. The main channel was subsequently coated with fibronectin (100 µg mL^−1^) for 1 h at 37 °C to promote endothelial cell attachment.

### Cell Culture and Microfluidic Seeding

Human breast adenocarcinoma cells (MCF‐7 and MDA‐MB‐231) were obtained from ATCC and maintained in DMEM supplemented with 10% FBS and 1% penicillin/streptomycin. Human Umbilical Vein Endothelial Cells (HUVECs, passage 3–6) were cultured in an endothelial growth medium (EGM‐2, Lonza). All cells were maintained at 37 °C with 5% CO_2_ and passaged at 80% confluence. Cells were harvested from devices using TrypLE treatment for 5 min before seeding.

Cancer cell seeding was performed using a mixture of cell suspension (1 × 10^6^ cells mL^−1^) and Matrigel (2:1 volume ratio). Following vacuum treatment (> 30 min), the mixture was introduced into niche chambers and centrifuged (2000 rpm, 30 s). After 15 min incubation on ice and 30 min incubation at 37 °C, a second Matrigel (mixed with a medium at 1:1 volume ratio) layer was added to seal the neck region. Similar to our previous works,^[^ ^5^, ^55^ ^]^ HUVECs (1 × 10^7^ cells mL^−1^) were seeded into the fibronectin‐coated main channel and allowed to attach for 30 min under static conditions. The microchip was turned over to facilitate both sides of the attachment. Dynamic culture was maintained using a syringe pump delivering culture medium at 1.875 µL min^−1^ (corresponding to 0.5 dyne cm^−^^2^ shear stress).

### Live imaging staining

For fluorescent live imaging, cancer cells were stained with PKH26 Red Fluorescent Cell Linker Kit and HUVECs with PKH67 Green Fluorescent Cell Linker Kit (Sigma‐Aldrich). Both cancer cells and HUVECs were stained with BioTracker TiY Vimentin Live Cell Dye (Sigma‐Aldrich) following manufacturer's protocols. Matrigel (Growth Factor Reduced, Corning) was mixed with cancer cell suspension at 1:2 volume ratio before seeding. Fibronectin solution (100 µg mL^−1^ in PBS) was prepared fresh for each experiment. Fluorescein isothiocyanatedextran (FITC‐Dex, 3‐ to 5‐kDa, Sigma‐Aldrich) was prepared fresh at concentration of 1 mg mL^−1^ for diffusion experiment. To examine the endothelium functionality, phorbol‐12‐myristate‐13‐acetate (PMA) with concentration of 50 ng mL^−1^ in serum‐free medium EGM^−2^ was added into the channel and incubated at 37 °C for 1.5 h before imaging.

### Immunofluorescence Staining

Cells were fixed with 4% paraformaldehyde for 1 h and then washed with PBS for 1 h. Cells were then permeabilized with 1% Triton X‐100 for 2 h and blocked with 3% BSA overnight in 4 °C fridge. Primary antibodies with fluorescence against CD31 (1:400), VE‐cadherin (1:400), Vimentin (1:3000), EpCAM (1:400), and Hoechst 33342 (1:1000) were incubated on a shaker for 3 days at room temperature. Afterward, PBS was added to wash the channel on a shaker for 2 days. To investigate ICAM‐1 expression in the main channel, cells were fixed with 4% paraformaldehyde for 10 min and then incubated with CD31 (1:300) and ICAM‐1 (1:200) for 1 h at 37 °C.

### Image Acquisition and Analysis

Time‐lapse imaging was performed using an environmentally controlled confocal microscope (Olympus FV3000RS) equipped with a 20× objective (NA 0.8). For z‐stack imaging to validate endothelial barrier integrity, confocal images were captured at different heights (50, 100, 150, and 200 µm) along the z‐axis using a 20× objective (NA 0.8). For endpoint analyses, high‐resolution confocal images were captured with a 2 µm z‐step size. FITC‐Dextran diffusion was imaged using the epifluorescence mode with a 20× objective at 30 and 60 s after injection. Image analysis was performed using ImageJ/FIJI software. Cell tracking was conducted using the Imaris (Bitplane AG, Oxford Instruments). Cell morphology parameters were analyzed using ImageJ/FIJI software. Fluorescence intensity profiles were generated using the Plot Profile function in ImageJ.

### Statistical Analysis

All experiments were performed with at least three biological replicates. Statistical analysis was performed using Origin software (OriginPro 2024). One‐Way ANOVA and Two‐Way ANOVA with Fishers Least Significant Difference (LSD) test was used for multiple comparisons. Data are presented as mean ± SEM of *n* ≥ 3, with *p* < 0.05 considered statistically significant.

## Conflict of Interest

The authors declare no conflict of interest.

## Author Contributions

F.J., H.L., and L.A.J. proposed research ideas and designed. F.J. performed the experiments, and established the INVADE platform, analyzed the data and prepared the manuscript. Y.Z. performed immunostaining and analysis. G.F., Y.W., Y.S., Y. W., K.S., Y.S.Z., A.C., and H.L. provided intellectual contribution to the tissue engineering in INVADE system along with critical suggestions to the technology development. J.J. and A.D. provided tissue culture support and quality controls. L.A.J. is the senior author who supervised the study and provided instruction, design suggestions for the improvement of the experiments. All authors provided comments and corrections to the manuscript writing.

## Supporting information



Supporting Information

## Data Availability

The data that support the findings of this study are available from the corresponding author upon reasonable request.

## References

[1] C. L. Chaffer , R. A. Weinberg , Science 2011, 331, 1559. 21436443 10.1126/science.1203543

[2] K. E. de Visser , J. A. Joyce , Cancer Cell 2023, 41, 374. 36917948 10.1016/j.ccell.2023.02.016

[3] N. Reymond , B. B. Agua d′ , A. J. Ridley , Nat. Rev. Cancer 2013, 13, 858. 24263189 10.1038/nrc3628

[4] I. K. Zervantonakis , S. K. Hughes‐Alford , J. L. Charest , J. S. Condeelis , F. B. Gertler , R. D. Kamm , Proc Natl Acad Sci U S A 2012, 109, 13515. 22869695 10.1073/pnas.1210182109 PMC3427099

[5] Y. Zhang , F. Jiang , Y. C. Zhao , A. N. Cho , G. Fang , C. D. Cox , H. Zreiqat , Z. F. Lu , H. Lu , L. A. Ju , Biomed. Mater. 2023, 18, 055008. 10.1088/1748-605X/ace7a4 37451254

[6] H.‐W. Cheng , Y.‐F. Chen , J.‐M. Wong , C.‐W. Weng , H.‐Y. Chen , S.‐L. Yu , H.‐W. Chen , A. Yuan , J. J. W. Chen , J. Exper. Clin. Cancer Res. 2017, 36, 27. 28173828 10.1186/s13046-017-0495-3 PMC5296960

[7] A. Lainé , O. Labiad , H. Hernandez‐Vargas , S. This , A. Sanlaville , S. Léon , S. Dalle , D. Sheppard , M. A. Travis , H. Paidassi , J. C. Marie , Nat. Commun. 2021, 12, 6228. 34711823 10.1038/s41467-021-26352-2 PMC8553942

[8] K. Jiang , S. B. Lim , J. Xiao , D. S. Jokhun , M. Shang , X. Song , P. Zhang , L. Liang , B. C. Low , G. V. Shivashankar , C. T. Lim , Adv. Sci. (Weinh) 2023, 10, 2201663. 37218524 10.1002/advs.202201663 PMC10401173

[9] W. J. Polacheck , A. E. German , A. Mammoto , D. E. Ingber , R. D. Kamm , Proc Natl Acad Sci U S A 2014, 111, 2447. 24550267 10.1073/pnas.1316848111 PMC3932905

[10] X. C. Li , J. L. Wang , Int. J. Biol. Sciences 2020, 16, 2014. 10.7150/ijbs.44943 PMC7294938 32549750

[11] G. Follain , D. Herrmann , S. Harlepp , V. Hyenne , N. Osmani , S. C. Warren , P. Timpson , J. G. Goetz , Nat. Rev. Cancer 2020, 20, 107. 31780785 10.1038/s41568-019-0221-x

[12] F. Font‐Clos , S. Zapperi , C. A. M. La Porta , iScience 2020, 23, 101073. 32361595 10.1016/j.isci.2020.101073 PMC7200936

[13] N. Rahmati , N. Maftoon , Front. Bioeng. Biotechnol. 2024, 12. 10.3389/fbioe.2024.1393413 PMC11163055 38860135

[14] D. X. Nguyen , P. D. Bos , J. Massague , Nat. Rev. Cancer 2009, 9, 274. 19308067 10.1038/nrc2622

[15] M. Park , D. Kim , S. Ko , A. Kim , K. Mo , H. Yoon , Int. J. Mol. Sci. 2022, 23, 6806. 35743249 10.3390/ijms23126806 PMC9224686

[16] K. Grubczak , A. Kretowska‐Grunwald , D. Groth , I. Poplawska , A. Eljaszewicz , L. Bolkun , A. Starosz , J. M. Holl , M. Mysliwiec , J. Kruszewska , M. Z. Wojtukiewicz , M. Moniuszko , Cells 2021, 10, 2044. 34440813 10.3390/cells10082044 PMC8392578

[17] S. Bersini , J. S. Jeon , M. Moretti , R. D. Kamm , Drug Discov. Today 2014, 19, 735. 24361339 10.1016/j.drudis.2013.12.006 PMC4048792

[18] K. E. Sung , D. J. Beebe , Adv Drug Deliv. Rev. 2014, 79–80, 68. 10.1016/j.addr.2014.07.002 PMC4258433 25017040

[19] S. N. Bhatia , D. E. Ingber , Nat. Biotechnol. 2014, 32, 760. 25093883 10.1038/nbt.2989

[20] M. B. Chen , J. A. Whisler , J. Frose , C. Yu , Y. Shin , R. D. Kamm , Nat. Protoc. 2017, 12, 865. 28358393 10.1038/nprot.2017.018 PMC5509465

[21] X. Shen , C. Wang , H. Zhu , Y. Wang , X. Wang , X. Cheng , W. Ge , W. Lu , J. Ovarian. Res. 2021, 14, 38. 33627162 10.1186/s13048-021-00776-2 PMC7905574

[22] J. Li , Q. Guo , X. Lei , L. Zhang , C. Su , Y. Liu , W. Zhou , H. Chen , H. Wang , F. Wang , Y. Yan , J. Zhang , J. Cancer 2020, 11, 6348. 33033518 10.7150/jca.44431 PMC7532514

[23] R. Augustine , A. A. Zahid , F. Mraiche , K. Alam , A. E. Al Moustafa , A. Hasan , Pharm. Dev. Technol. 2021, 26, 490. 33416013 10.1080/10837450.2021.1872624

[24] M. Hakim , L. Kermanshah , H. Abouali , H. M. Hashemi , A. Yari , F. Khorasheh , I. Alemzadeh , M. Vossoughi , Biophys. Rev. 2022, 14, 517. 35528034 10.1007/s12551-022-00944-8 PMC9043145

[25] P. Mehta , Z. Rahman , P. ten Dijke , P. E. Boukany , Trends Cancer 2022, 8, 683. 35568647 10.1016/j.trecan.2022.03.006

[26] S. Bersini , J. S. Jeon , G. Dubini , C. Arrigoni , S. Chung , J. L. Charest , M. Moretti , R. D. Kamm , Biomaterials 2014, 35, 2454. 24388382 10.1016/j.biomaterials.2013.11.050 PMC3905838

[27] Y. Zhang , S. D. Z. Ramasundara , R. E. Preketes‐tardiani , V. Cheng , H. Lu , L. A. Ju , Frontiers in Cardiovascular Medicine 2021, 8, 766513. 34901226 10.3389/fcvm.2021.766513 PMC8655735

[28] I. Sigdel , N. Gupta , F. Faizee , V. M. Khare , A. K. Tiwari , Y. Tang , Front. Bioeng. Biotechnol. 2021, 9, 633671. 33777909 10.3389/fbioe.2021.633671 PMC7992012

[29] S. Devarasou , M. Kang , J. H. Shin , APL Bioeng. 2024, 8, 021507. 38855445 10.1063/5.0199024 PMC11161195

[30] V. Azimian Zavareh , L. Rafiee , M. Sheikholeslam , L. Shariati , G. Vaseghi , H. Savoji , S. Haghjooy Javanmard , ACS Biomater. Sci. Eng. 2022, 8, 4648. 36260561 10.1021/acsbiomaterials.2c00277

[31] F. Paradiso , S. Serpelloni , L. W. Francis , F. Taraballi , Int. J. Mol. Sci. 2021, 22, 10098. 34576261 10.3390/ijms221810098 PMC8472581

[32] C. D. Paul , P. Mistriotis , K. Konstantopoulos , Nat. Rev. Cancer 2017, 17, 131. 27909339 10.1038/nrc.2016.123 PMC5364498

[33] B. Strilic , S. Offermanns , Cancer Cell 2017, 32, 282. 28898694 10.1016/j.ccell.2017.07.001

[34] M. K. Sznurkowska , N. Aceto , FEBS J. 2022, 289, 4336. 34077633 10.1111/febs.16046 PMC9546053

[35] A. Nurmagambetova , V. Mustyatsa , A. Saidova , I. Vorobjev , Sci. Rep. 2023, 13, 22164. 38092761 10.1038/s41598-023-48279-y PMC10719275

[36] H. T. Morris , L. M. Machesky , Br. J. Cancer 2015, 112, 613. 25611303 10.1038/bjc.2014.658 PMC4333498

[37] C. T. Mierke , Rep. Prog. Phys. 2019, 82, 064602. 30947151 10.1088/1361-6633/ab1628

[38] C. Z. Eddy , H. Raposo , A. Manchanda , R. Wong , F. Li , B. Sun , Sci. Rep. 2021, 11, 20434. 34650167 10.1038/s41598-021-99902-9 PMC8516896

[39] J. Fares , M. Y. Fares , H. H. Khachfe , H. A. Salhab , Y. Fares , Signal Transduct Target Ther 2020, 5, 28. 32296047 10.1038/s41392-020-0134-x PMC7067809

[40] A. Martowicz , G. Spizzo , G. Gastl , G. Untergasser , BMC Cancer 2012, 12, 501. 23110550 10.1186/1471-2407-12-501 PMC3519683

[41] T. Tonini , F. Rossi , P. P. Claudio , Oncogene 2003, 22, 6549. 14528279 10.1038/sj.onc.1206816

[42] P. Carmeliet , R. K. Jain , Nature 2000, 407, 249. 11001068 10.1038/35025220

[43] B. K. Ahir , H. H. Engelhard , S. S. Lakka , Mol. Neurobiol. 2020, 57, 2461. 32152825 10.1007/s12035-020-01892-8 PMC7170819

[44] T. Annese , R. Tamma , S. Ruggieri , D. Ribatti , Cancers (Basel) 2019, 11, 381. 30889903 10.3390/cancers11030381 PMC6468440

[45] E. J. Sohn , D. B. Jung , H. Lee , I. Han , J. Lee , H. Lee , S. H. Kim , Cancer Lett. 2018, 412, 88. 29024811 10.1016/j.canlet.2017.09.052

[46] Y. Kawai , M. Kaidoh , T. Ohhashi , Am. J. Physiol. Cell Physiol. 2008, 295, C1123. 18768924 10.1152/ajpcell.00247.2008

[47] L. Wang , D. D. Du , Z. X. Zheng , P. F. Shang , X. X. Yang , C. Sun , X. Y. Wang , Y. J. Tang , X. L. Guo , Front. Pharmacol. 2022, 13, 979474. 36386163 10.3389/fphar.2022.979474 PMC9642840

[48] S. M. Weis , D. A. Cheresh , Nat. Cell Biol. 2013, 15, 721. 23817234 10.1038/ncb2794

[49] J. W. Song , L. L. Munn , Proc. Natl Acad. Sci. USA 2011, 108, 15342. 21876168 10.1073/pnas.1105316108 PMC3174629

[50] D. Wirtz , K. Konstantopoulos , P. C. Searson , Nat. Rev. Cancer 2011, 11, 512. 21701513 10.1038/nrc3080 PMC3262453

[51] N. Osmani , G. Follain , M. J. G. León , O. Lefebvre , I. Busnelli , A. Larnicol , S. Harlepp , J. G. Goetz , Cell Rep. 2019, 28, 2491. 31484062 10.1016/j.celrep.2019.07.102

[52] A. Haeger , M. Krause , K. Wolf , P. Friedl , Biochim. Biophys. Acta. 2014, 1840, 2386. 24721714 10.1016/j.bbagen.2014.03.020

[53] L. A. Ju , S. Kossmann , Y. C. Zhao , L. Moldovan , Y. Zhang , S. De Zoysa Ramasundara , F. Zhou , H. Lu , I. Alwis , S. M. Schoenwaelder , Y. Yuan , S. P. Jackson , The Analyst 2022, 147, 1222. 35212697 10.1039/d2an00270a

[54] G. Fang , H. Lu , R. Al‐Nakashli , R. Chapman , Y. Zhang , L. A. Ju , G. Lin , M. H. Stenzel , D. Jin , Biofabrication 2021, 14, 015006. 10.1088/1758-5090/ac2ef9 34638112

[55] Y. Zhang , S. Aye , V. Cheng , A. Nasser , T. Hong , P. Vatankhah , F. Jiang , Y. C. Zhao , L. Moldovan , A. Sun , A. Dupuy , Y. Wang , Z. Li , T. Ang , F. Passam , K. Yong , L. A. Ju , Adv. Mater. Interfaces 2023, 10, 2300234.

