# Supplementary material for: Intravasation‐On‐µDevice (INVADE): Engineering Dynamic Vascular Interfaces to Study Cancer Cell Intravasation
Source: Adv Mater. 2025 Apr 13;37(26):2501466. doi: 10.1002/adma.202501466 (PMC12232236; doi:10.1002/adma.202501466)

**Supplementary Materials:**

**Supplementary Figure S1**, corresponding to Figure 1. Geometrical diagram of the INVADE platform showing the complete channel design with 23 parallel niche chambers. The main channel (width: 300 μm, height: 200 μm) connects to niche chambers (radius: 75 μm) via neck constrictions (width: 40 μm, length: 255 μm). This design enables high-throughput analysis of cancer-endothelial interactions under controlled conditions.
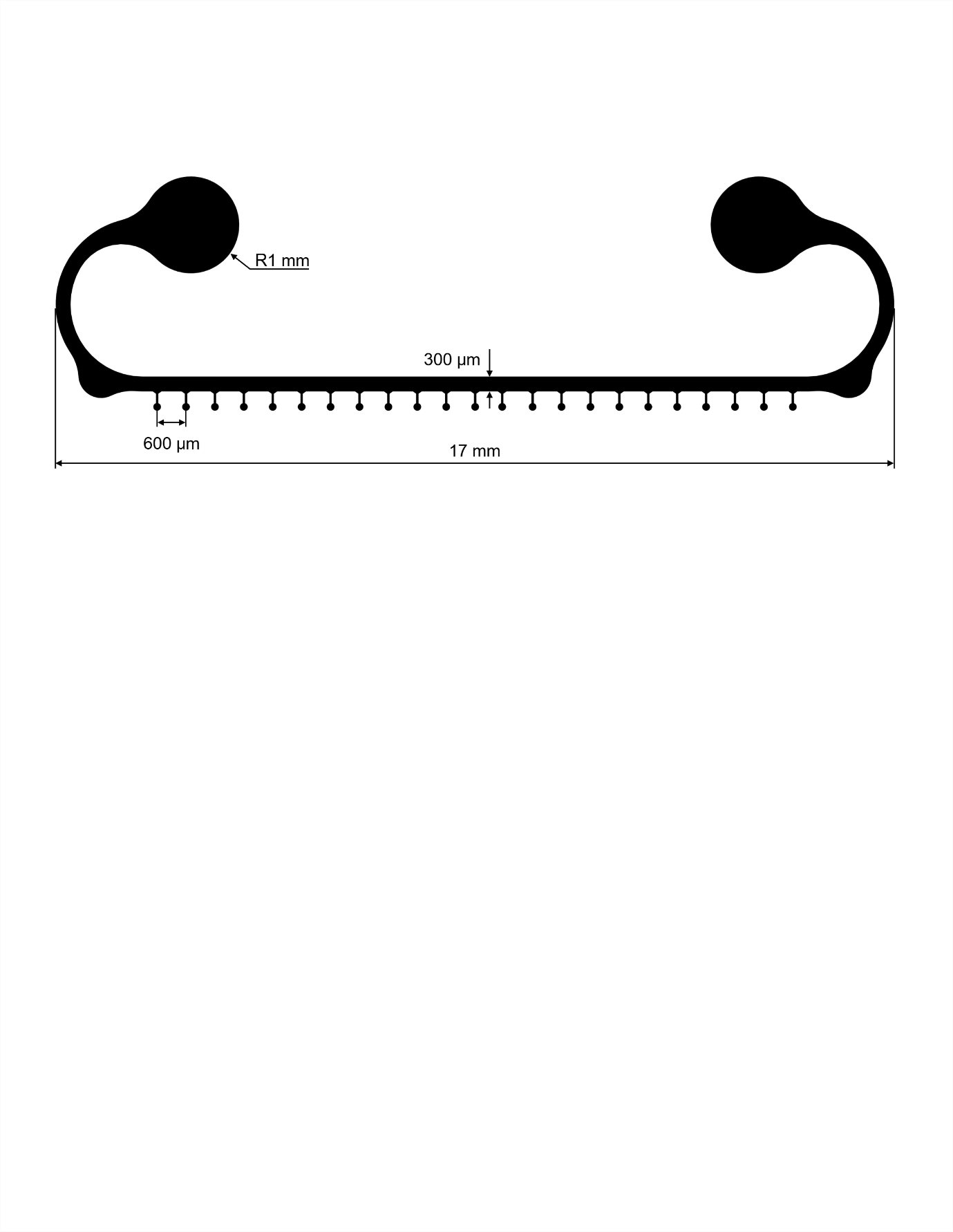


**Supplementary Figure S2**, corresponding to Figure 1. Photogram of INVADE PDMS microchip fabricated using standard soft lithography. The chip dimensions are 20 mm × 20 mm with a thickness of 3-5 mm. The microchannels were created using dry photoresist film on silicon wafers, followed by PDMS casting (10:1 base to curing agent ratio) and oxygen plasma bonding to PDMS membrane.
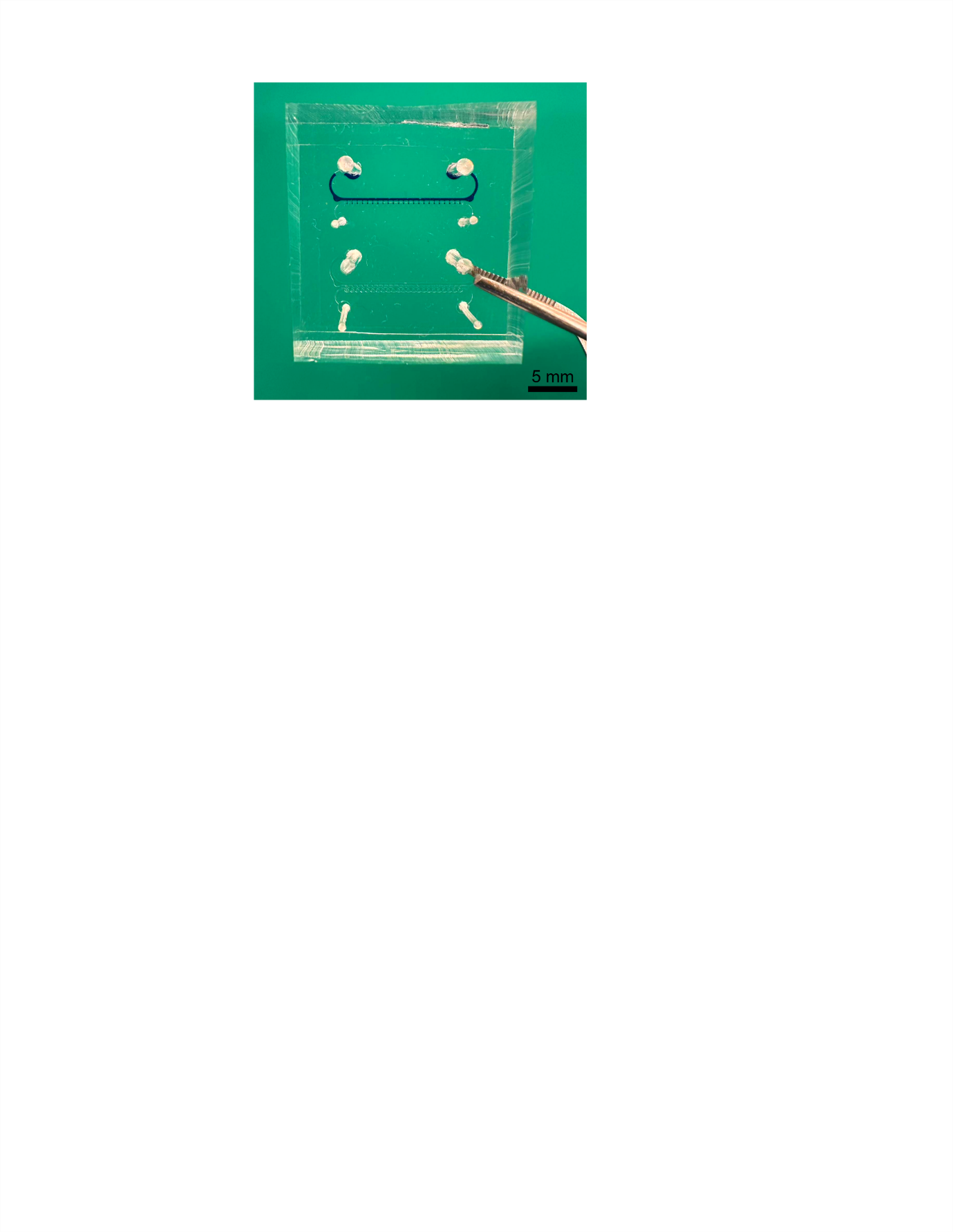


**Supplementary figure S3**, corresponding to Figure 2. Computational flow dynamics simulation of dynamic cultured INVADE platform performed using COMSOL Multiphysics 6.0. Flow rates of 1.875 μL min^-1^generate shear stresses of 0.501 dyne cm^-2^ in the main channel, with minimal flow penetration into the niche chambers. Color scale indicates velocity magnitude (10^-4^ m s^-1^). Simulations were conducted assuming laminar flow conditions with no-slip boundary conditions at walls.


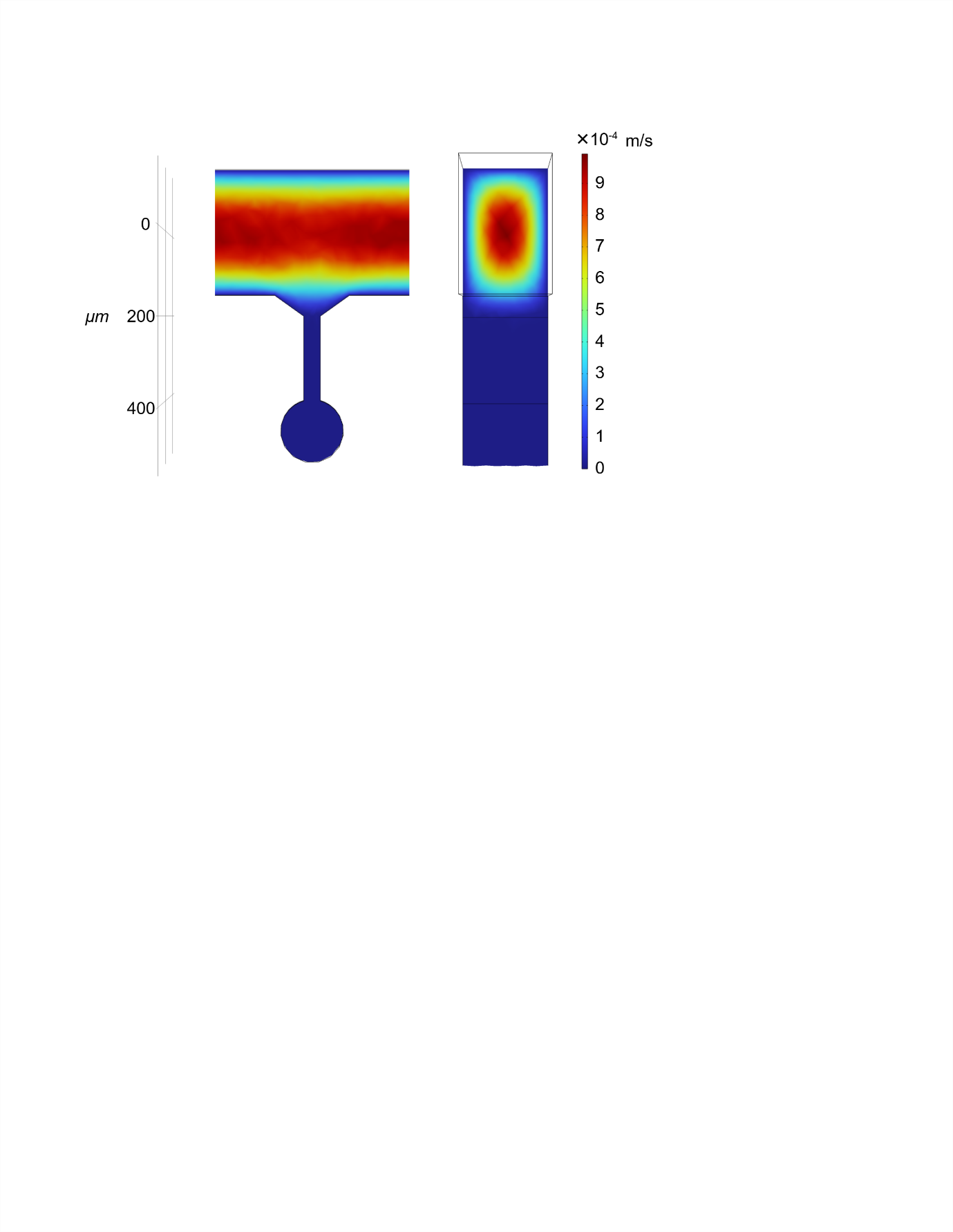


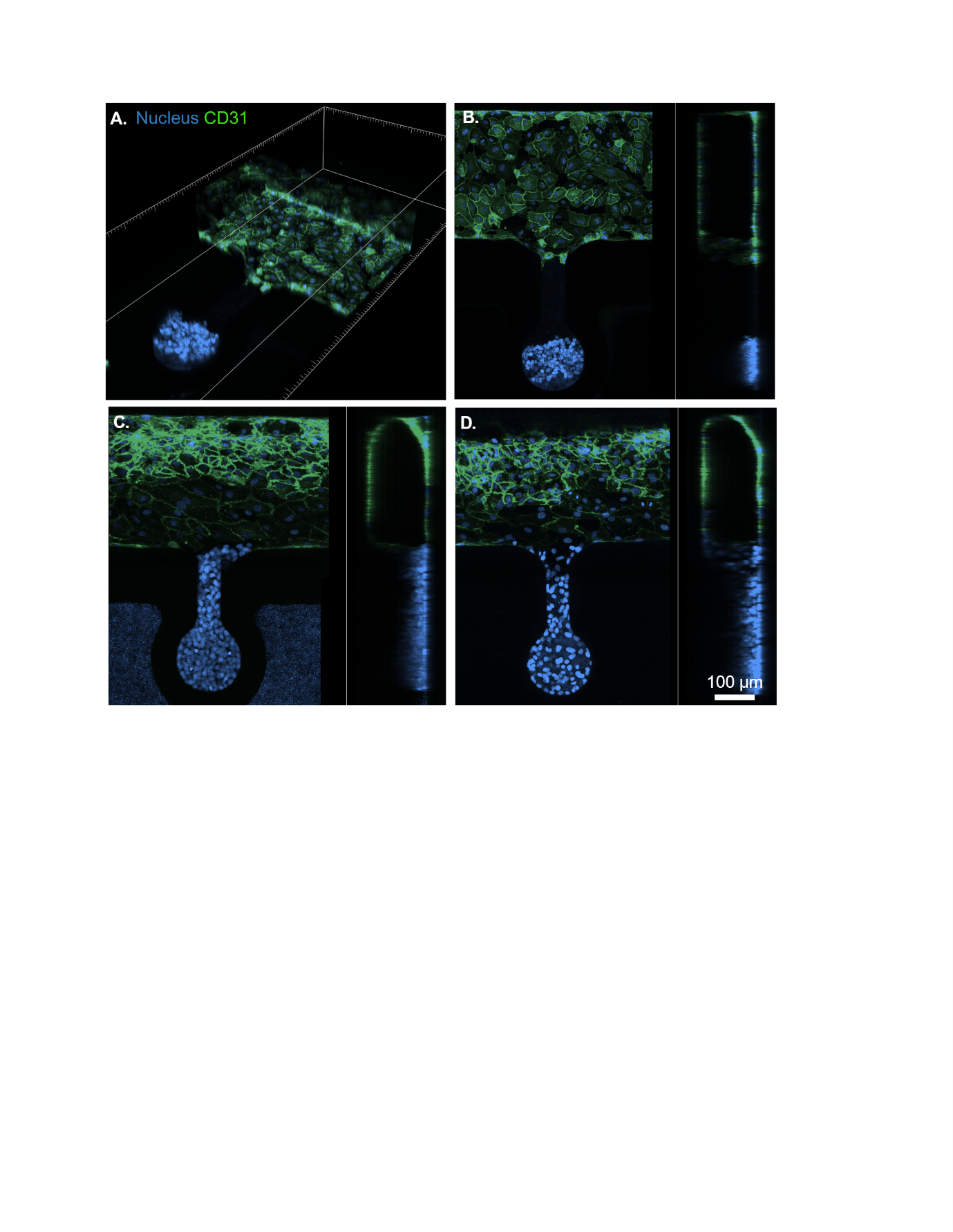
**Supplementary figure S4**. corresponding to Figure 2. Confocal images of 2-day cultured INVADE platform acquired using Olympus FV3000RS confocal microscope. A) 3D view and B) cross-section view of MCF-7+HUVECs at static cultured condition. C) Cross-section view of MCF-7+HUVECs at dynamic cultured condition (1.875 μL min^-1^). D) Cross-section view of MDA-MB-231+HUVECs at dynamic cultured condition (1.875 μL min^-1^). Images were captured with a 20× objective with 2 μm z-step size. Scale bars: 100 μm.

**Supplementary figure S5**, corresponding to Figure 2. Representative confocal images of the INVADE platform confirming intact endothelial barrier formation. Images were captured at cross-sectional planes along the z-axis at heights of 50, 100, 150, and 200 μm using Olympus FV3000RS confocal microscope. HUVECs were immunostained with CD31 (1:400, green), while nucleus of both HUVECs and MDA-MB-231 cells were labeled with Hoechst 33342 (1:1000, blue). Scale bar: 50 μm.


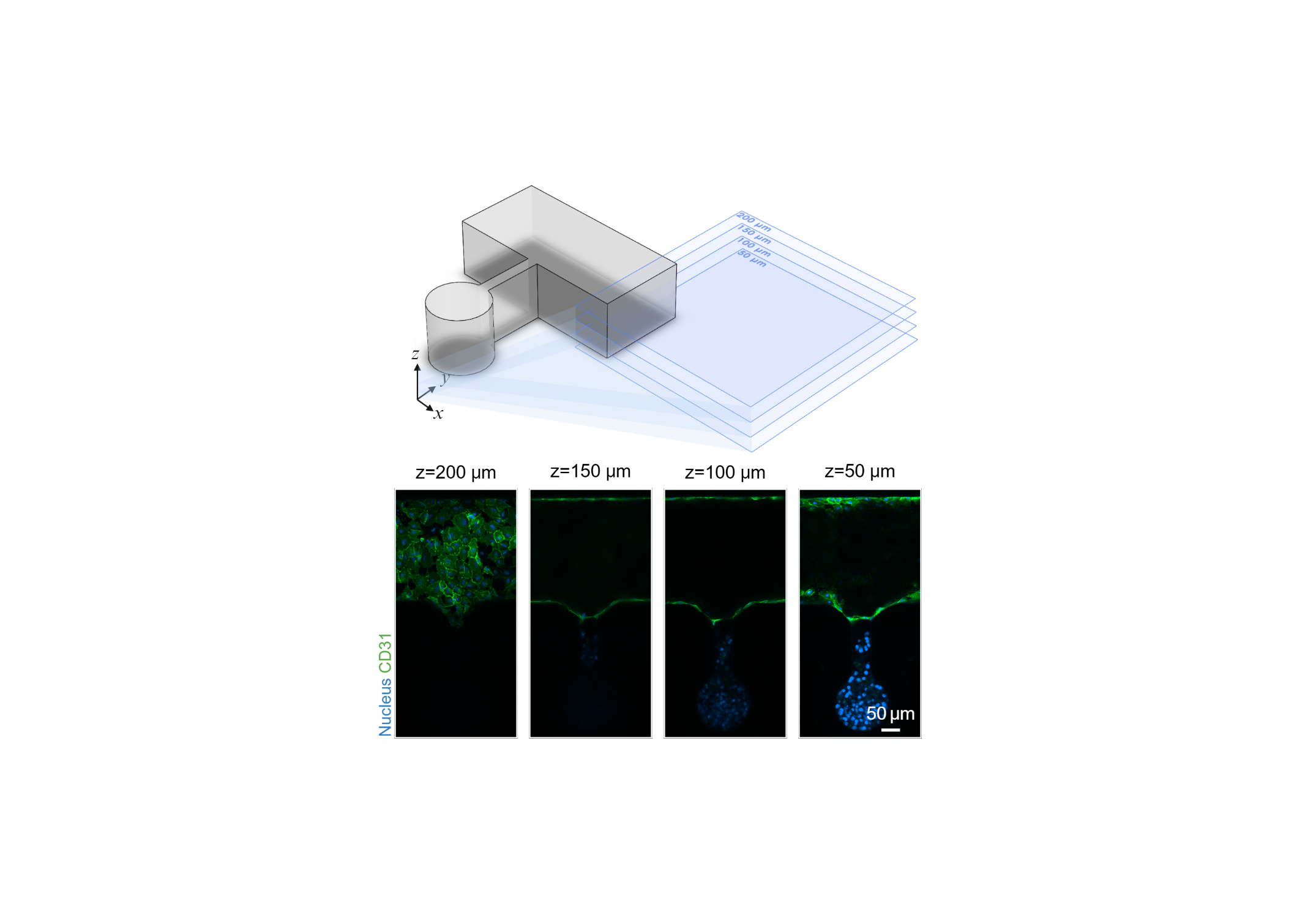


**Supplementary figure S6**, corresponding to Figure 2. Diffusion of 3- to 5-kDa FITC-Dextran (1 mg mL^-1^) in the INVADE platform demonstrating endothelial barrier functionality. Images show diffusion in the absence (left) and presence (middle and right) of an endothelium formed by HUVECs. In the right image, PMA was added at a concentration of 50 ng mL^-1^ for 1.5 h at 37 °C prior to imaging. Images were captured at 30 s and 60 s after FITC-Dex injection with a 20× objective. HUVECs were labeled with CD31 (1:300, green), and FITC-Dex was visualized in red. Scale bar: 50 μm.


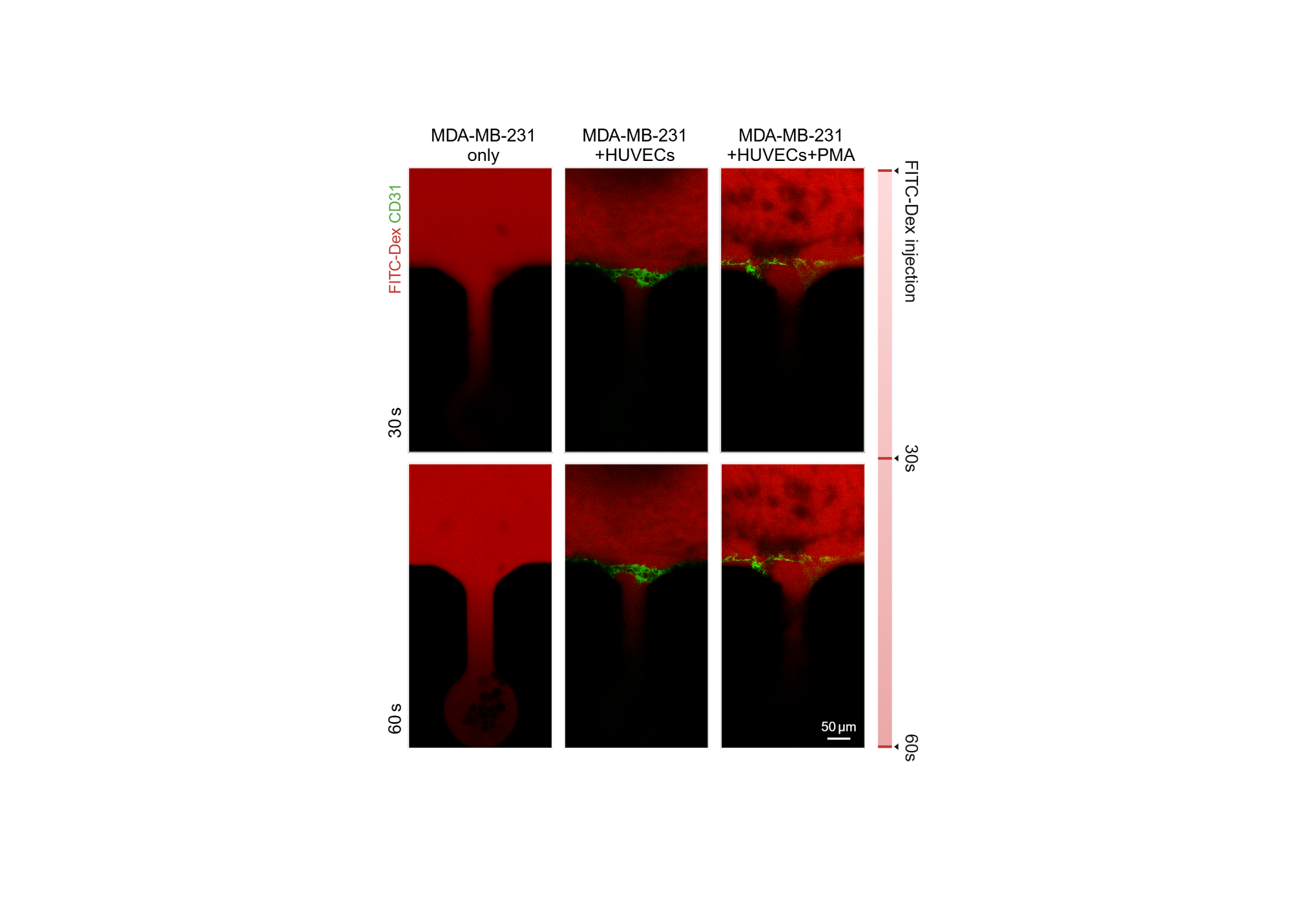


**Supplementary figure S7,** corresponding to Figure 5. Cancer-endothelial crosstalk promotes distinct intravasation patterns. A) Experimental setup: Four conditions (MCF-7 only, MDA-MB-231 only, MCF-7+HUVECs, MDA-MB-231+HUVECs) were tested in both static and dynamic culture systems (1.875 μL min^-1^) over two days, followed by confocal imaging and quantitative analysis. B) Immunofluorescence imaging demonstrates increased EMT marker expression and enhanced endothelial remodeling under flow conditions compared to static culture. Cells were stained for Vimentin (1:3000), EpCAM (1:400), and CD31 (1:400). Scale bar: 70 μm. C) Quantitative analysis of flow-dependent cancer cell proliferation. Data are presented as mean ± SEM. Statistical significance was assessed using Two-Way ANOVA (****p* < 0.001; n ≥ 3).


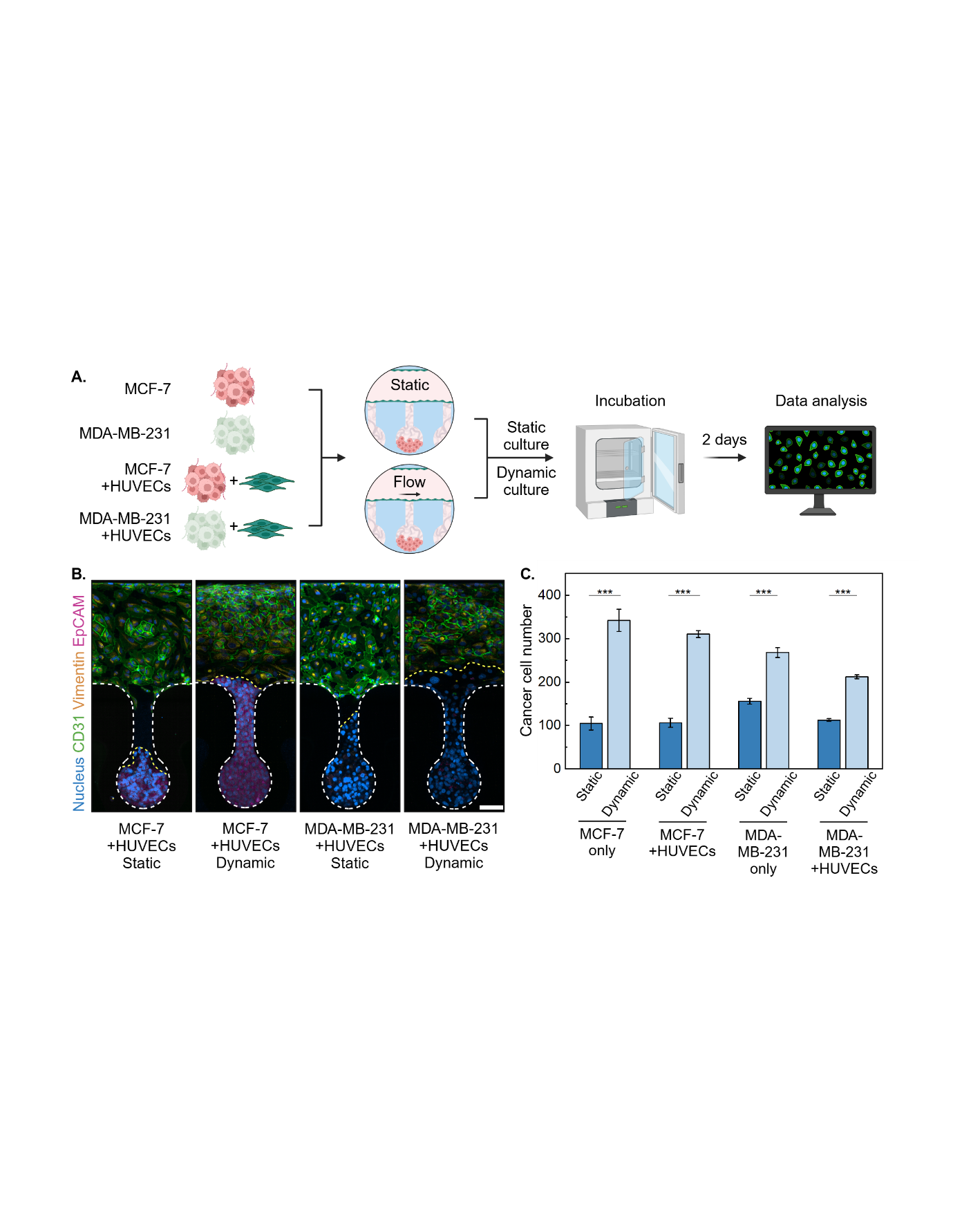


**
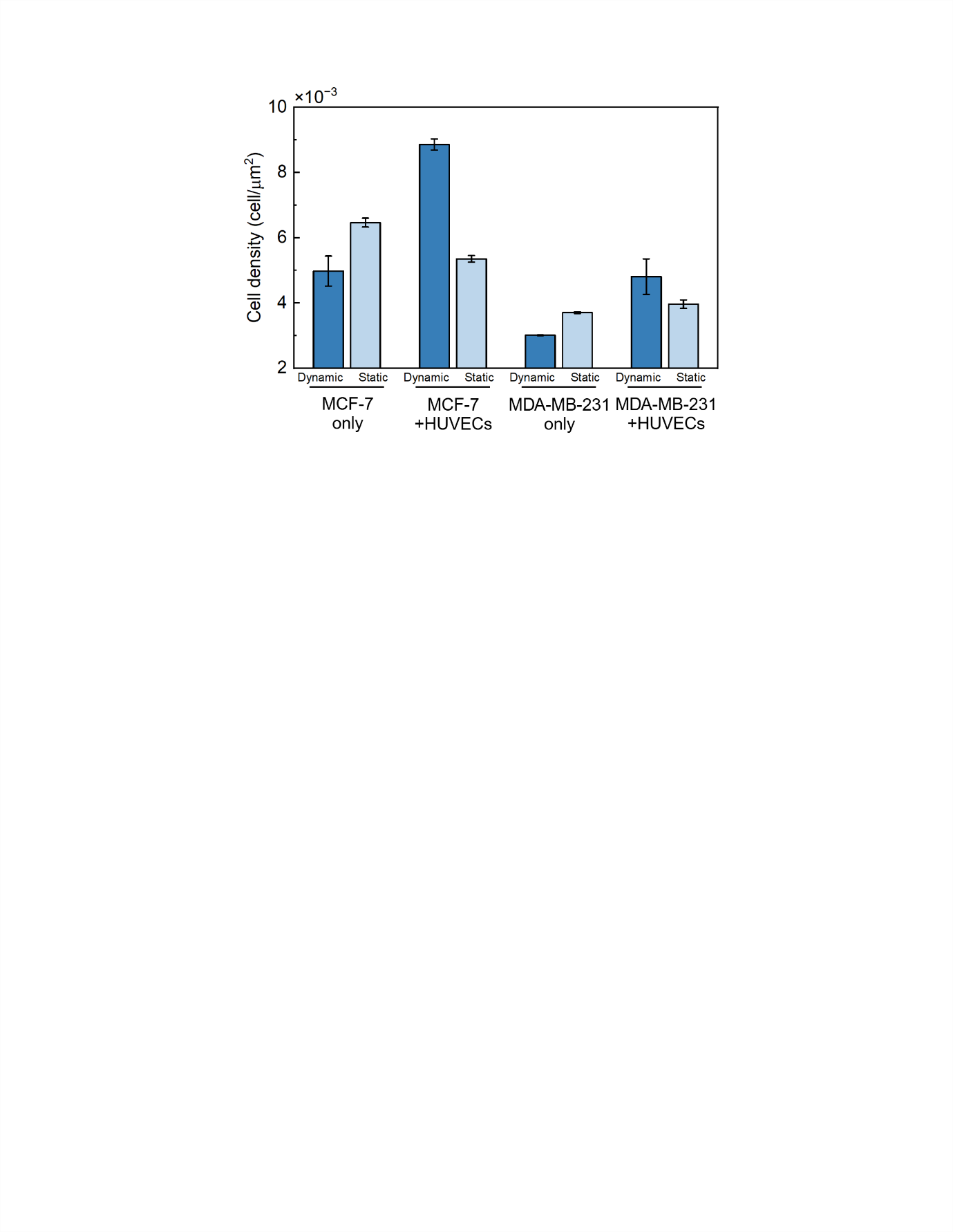
Supplementary figure S8,** corresponding to Figure 5. Quantitative analysis of flow-dependent cancer cell density in static versus dynamic (1.875 μL min^-1^) culture conditions. Cell density was calculated by dividing cell number by the area occupied within each niche chamber. Data are presented as mean ± SEM from at least 10 chambers per condition. Statistical significance was assessed using Two-Way ANOVA (****p* < 0.001; n ≥ 3).

**Supplementary figure S9**, corresponding to Figure 6. Confocal images of 2 independent samples showing cancer-endothelial cell interactions on day 4 for MDA-MB-231+HUVECs co-culture group. Images demonstrate epithelial-mesenchymal transition (EMT) markers (Vimentin (1:3000) and EpCAM (1:400)) and platelet endothelial cell adhesion molecule (CD31, 1:400, green). Red arrows indicate HUVECs that have migrated into the niche chambers, demonstrating bilateral cancer-endothelial interactions. Images were acquired using Olympus FV3000RS confocal microscope with a 20× objective. Scale bar: 80 μm.
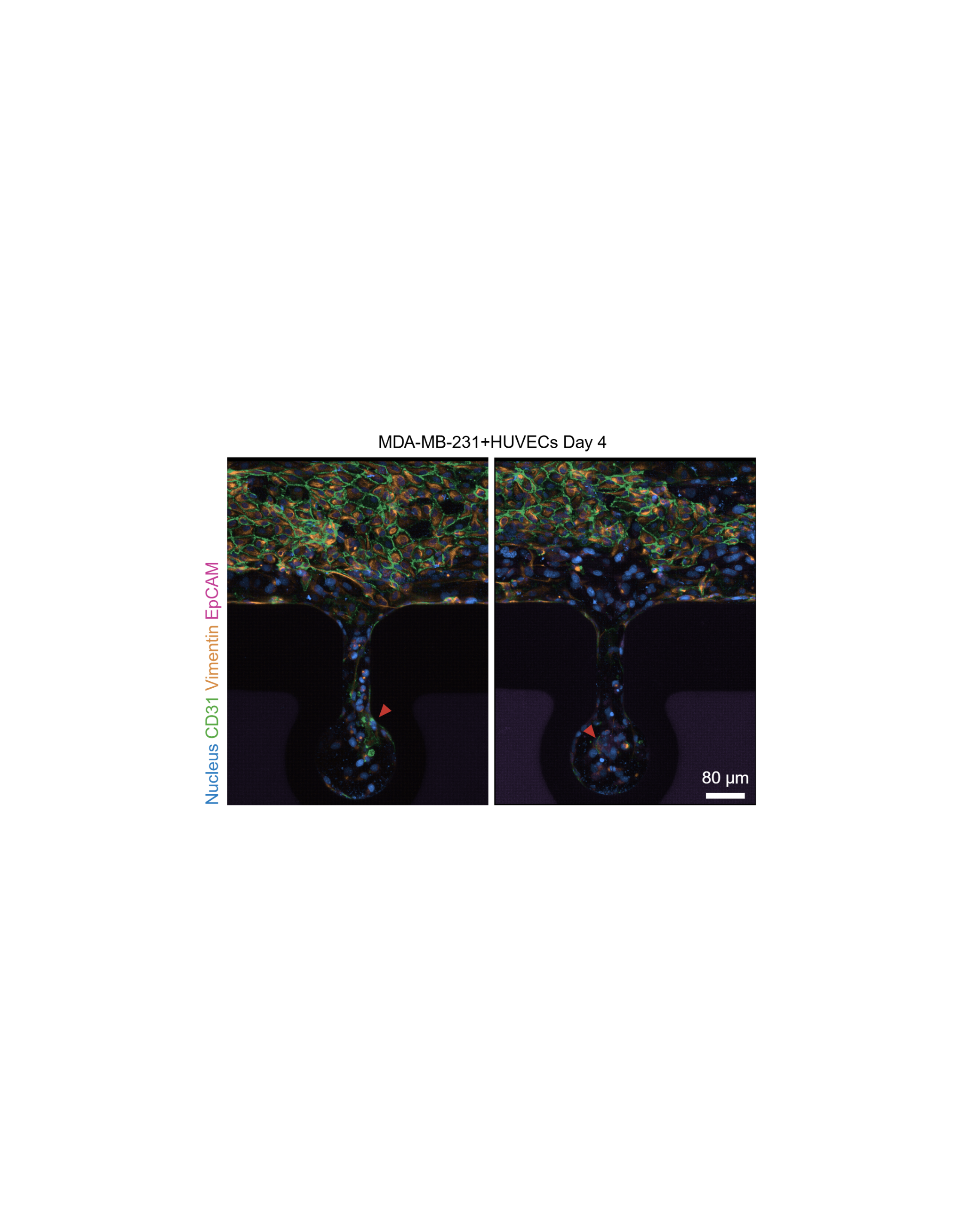


**Supplementary figure S10,** corresponding to Figure 7. Experimental setup for comparative analysis of endothelial remodeling in response to different cancer cell types. Two breast cancer cell lines, MCF-7 and MDA-MB-231, were cocultured with HUVECs for 48 h under dynamic conditions (1.875 μL min^-1^) prior to fixation, immunostaining, confocal imaging, and quantitative analysis.


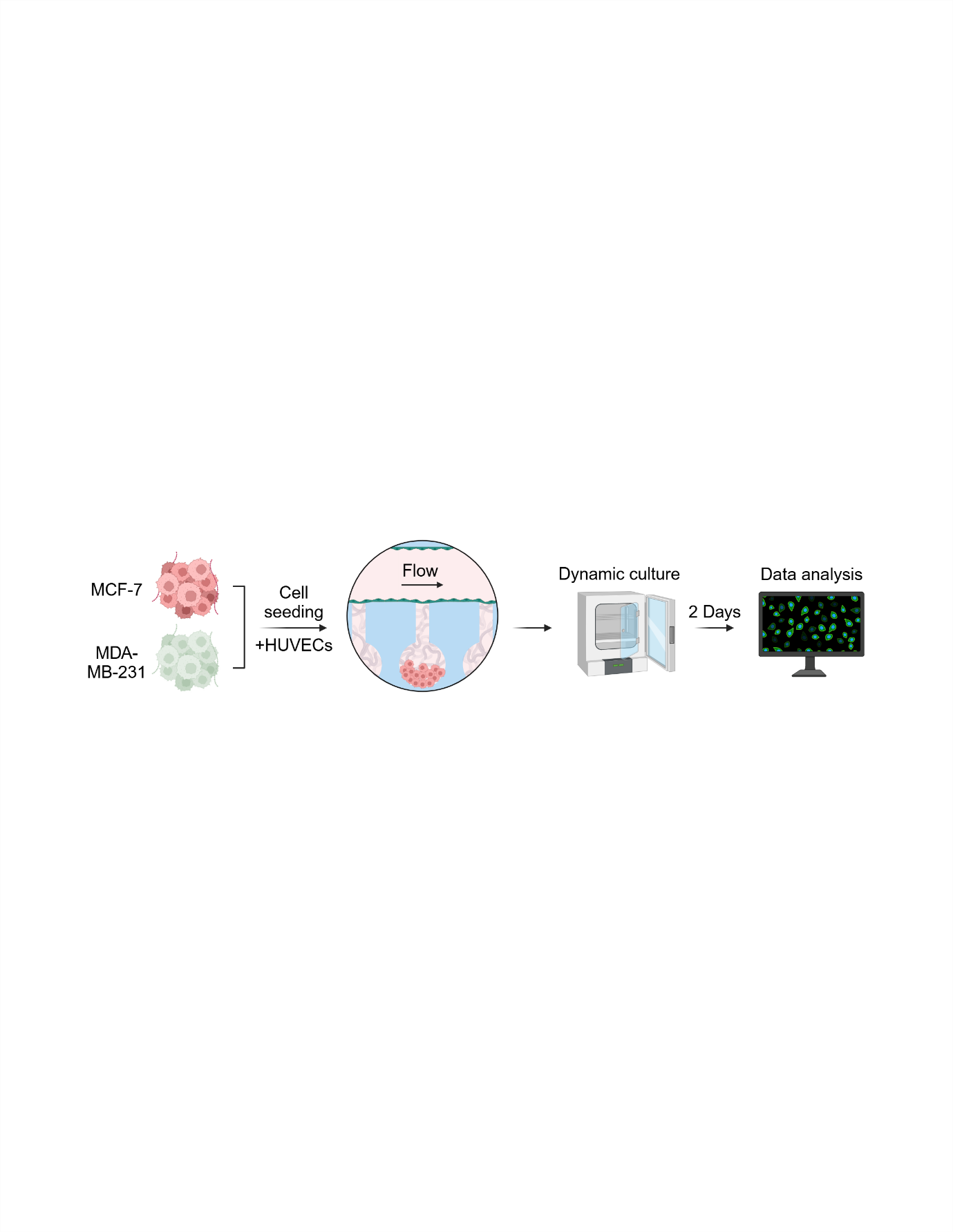


**
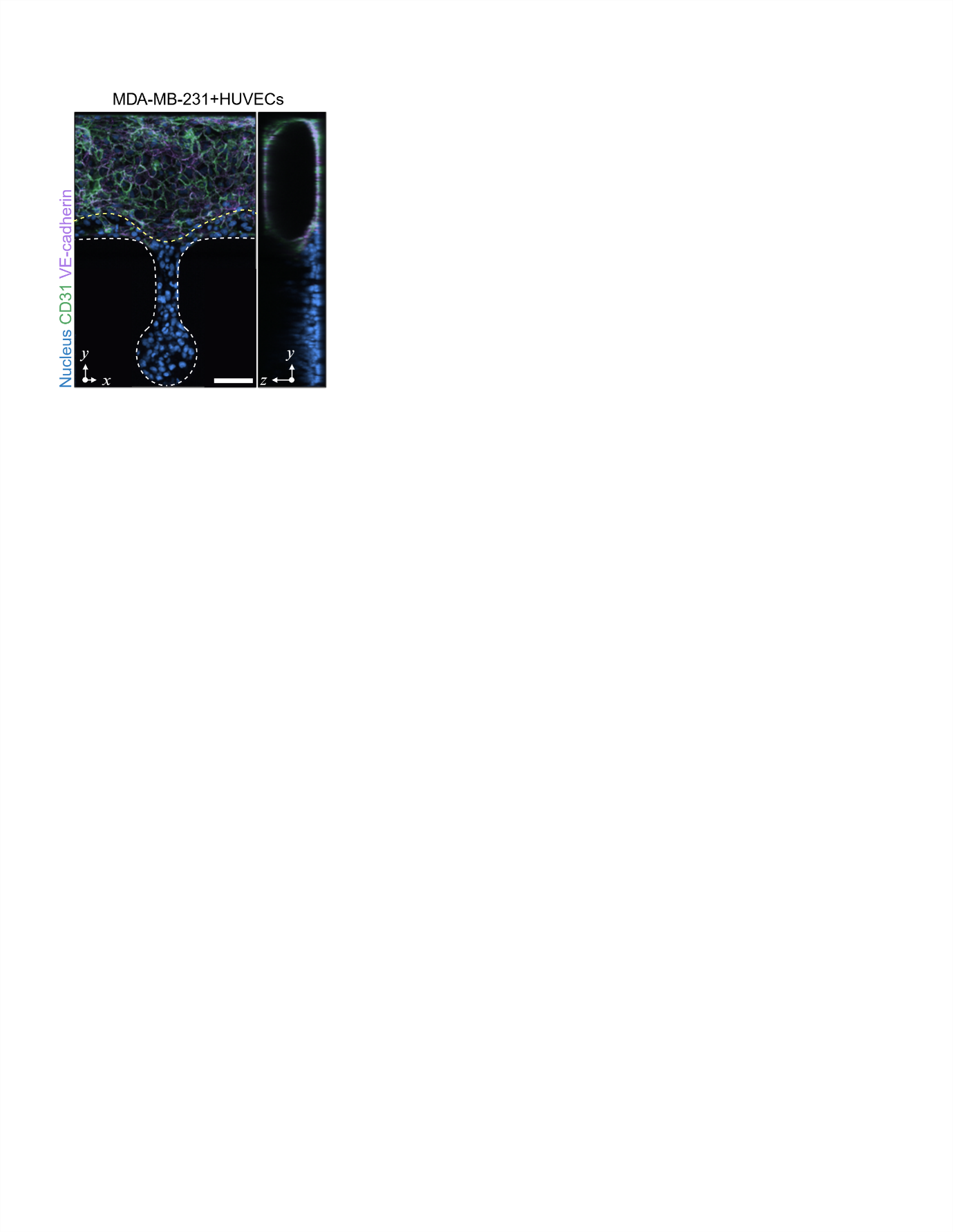
Supplementary figure S11,** corresponding to Figure 7. Spatial mapping of HUVECs and MDA-MB-231 cancer cells showing distinct remodeling patterns at the cancer-endothelial interface. Cells were fixed after 48 h of coculture under dynamic conditions (1.875 μL min^-1^) and stained for CD31 (1:300, green), VE-cadherin (1:400, purple), and nuclei (Hoechst 33342, 1:1000, blue). Images were acquired using Olympus FV3000RS confocal microscope with a 20× objective and processed for spatial analysis using ImageJ software. Scale bar: 100 μm.

**Supplementary figure S12,** corresponding to Figure 7. Quantitative analysis of endothelial remodeling. A) Comparative analysis of HUVECs aspect ratio (AR) distributions measured using automated cell segmentation in ImageJ (more than 30 cells per condition). B) CD31 intensity profile across the main channel width, expressed in arbitrary units (A.U.) measured using the Plot Profile function in ImageJ. Box plots in (A) indicate the median (middle line), mean (square), the first and third quartiles (box), and the 10th and 90th percentile (error bars) of the HUVECs AR. Statistical significance was determined by Two-Way ANOVA (**p* < 0.05; ***p* < 0.01; ****p* < 0.001; n ≥ 30).


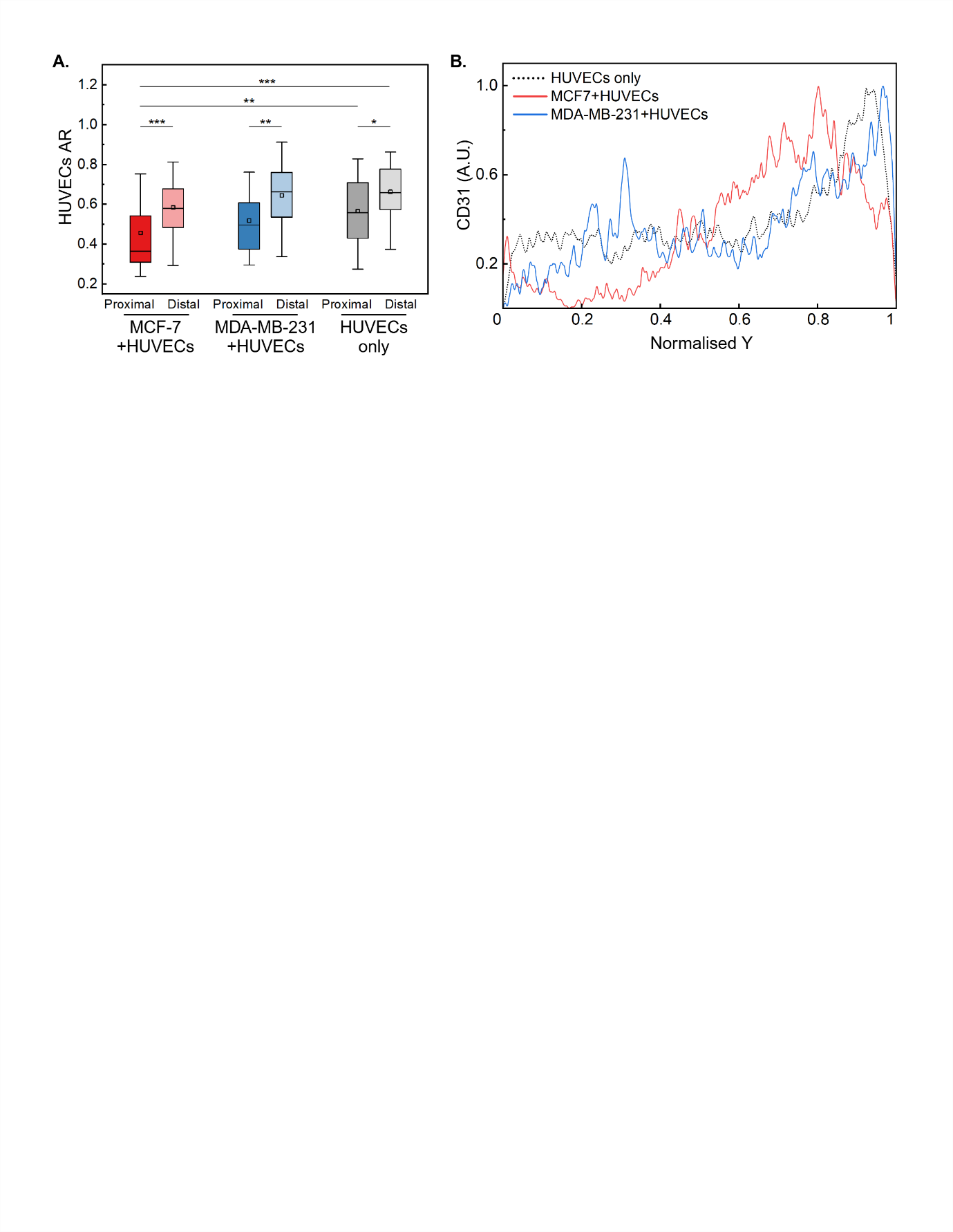

Supplement: Supplementary file 1 — Supporting Information [file ADMA-37-2501466-s001.docx]
